# Transcription Factor HOXC11 Drives Colorectal Cancer Progression and Metastasis via CAMK2A‐Dependent CXCL5 Upregulation

**DOI:** 10.1002/advs.76992

**Published:** 2026-08-19

**Authors:** Qingyang Sun, Hengjie Xu, Sheng Yang, Jiahui Zhou, Chuanxin Tian, Hongxu Nie, Chi Jin, Tuo Wang, Zhihao Chen, Junwei Tang, Yifei Feng, Xiaowei Wang, Yueming Sun

**Affiliations:** ^1^ Department of General Surgery Colorectal Institute of Nanjing Medical University The First Affiliated Hospital of Nanjing Medical University Nanjing China; ^2^ Jiangsu Province Engineering Research Center of Colorectal Cancer Precision Medicine and Translational Medicine Nanjing China; ^3^ Collaborative Innovation Center for Cancer Personalized Medicine Nanjing Medical University Nanjing China; ^4^ The Affiliated Shuyang Hospital of Xuzhou Medical University Suqian China; ^5^ Department of General Surgery The Third Affiliated Hospital of Nanjing Medical University Changzhou China; ^6^ Department of General Surgery Qilu Hospital of Shandong University Jinan China

**Keywords:** CAMK2A, colorectal cancer, CXCL5, HOXC11, NF‐κB

## Abstract

Colorectal cancer (CRC) remains a leading cause of cancer mortality, and the molecular drivers of progression and distant metastasis are incompletely understood. By integrating differential expression and survival analyses of TCGA CRC cohorts with HOX family genes, we identified HOXC11 as a key metastasis‐associated factor. HOXC11 was markedly upregulated in CRC tissues and cell lines, with higher expression in metastatic lesions than in primary tumors, and elevated HOXC11 correlated with poor patient prognosis. HOXC11 functionally increased CRC cell proliferation, migration, and invasion in vitro and facilitated tumor growth and metastasis in vivo. Mechanistically, HOXC11 directly bound to the CAMK2A promoter and transactivated CAMK2A, leading to increased phosphorylated CAMK2A and initiation of the NF‐κB pathway, which facilitated p65 nuclear translocation and induced CXCL5 expression to drive CRC progression. Conversely, CXCL5 signaling through CXCR2 upregulated HOXC11 via the ERK1/2‐SP1 axis, forming a positive feedback loop. Notably, combined inhibition of CAMK2A (KN‐93) and CXCR2 (SB265610) significantly attenuated HOXC11‐mediated proliferation and metastasis. Collectively, these findings define a HOXC11–CAMK2A–NF‐κB–CXCL5 circuit as a potential therapeutic target in CRC.

## Graphical abstract

HOXC11 drives colorectal cancer progression by transcriptionally activating CAMK2A, which triggers NF‐κB–dependent CXCL5 upregulation. CXCL5–CXCR2 signaling further reinforces HOXC11 expression through the ERK1/2–SP1 axis, forming a prometastatic positive feedback loop that is effectively disrupted by combined CAMK2A and CXCR2 inhibition.

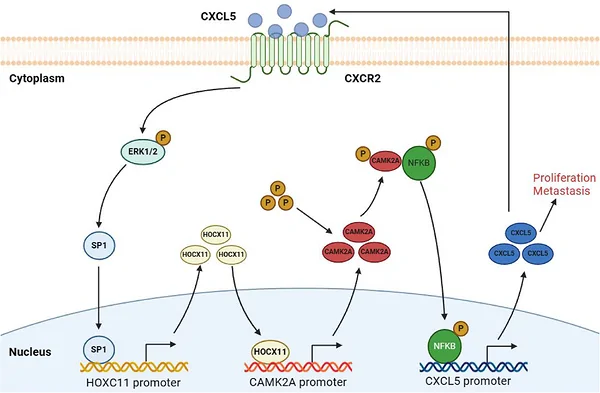

## 1 Introduction

Colorectal cancer (CRC) is considered one of the most common malignancies worldwide. According to the latest global cancer statistics from 2025, CRC ranks third in incidence and second in mortality [1]. It has been reported that in 2020, the estimated number of newly diagnosed CRC cases exceeded 1.9 million, and the estimated number of deaths exceeded 935,000 [2]. Surgical resection and radiotherapy/chemotherapy remain the mainstay treatments for CRC. Although recent advances in immunotherapy and targeted therapy have markedly improved therapeutic outcomes, the prognosis of CRC patients—particularly those with advanced disease—remains poor [3]. Therefore, identifying key targets and delineating the molecular mechanisms driving CRC progression is of great importance for developing new therapeutic strategies.

The homeobox (HOX) gene family comprises a group of transcription factors characterized by a highly conserved homeodomain and serves as a key regulator of embryonic development and diverse physiological processes, playing crucial roles in cell growth, differentiation, apoptosis, angiogenesis, and receptor‐mediated signal transduction [4]. In humans, the HOX family consists of 39 genes organized into four distinct clusters (HOXA, HOXB, HOXC, and HOXD) [5]. Accumulating evidence indicates that HOX genes are critically involved in tumor initiation, progression, and metastasis [4, 6, 7]. For example, HOXA1 acts as an oncogene to promote aerobic glycolysis in cervical cancer, thereby facilitating tumor progression [8]. In bladder cancer, HOXA1 enhances SMAD3 transcription to drive proliferation and metastasis [9], and its dysregulation is also associated with poor prognosis in breast and gastric cancers [10, 11]. HOXB5 has likewise been linked to cancer progression and metastasis in multiple malignancies, including prostate [12], pancreatic [13], breast [14], esophageal [15], and gastric cancer [16]. By contrast, HOXA5 has been identified as a tumor suppressor in cancers [17, 18]. Notably, HOXB13 has been reported to exert context‐dependent functions across tumor types: it promotes migration and invasion of gastric cancer cells by activating the PI3K/AKT/mTOR pathway [19], yet acts as a tumor suppressor in right‐sided colon cancer [20]. HOXC11, a member of the HOXC subfamily, has recently attracted increasing attention as a gene aberrantly expressed in diverse malignancies and potentially implicated in tumor progression [21, 22, 23]. Mechanistically, HOXC11 has been reported to drive lung adenocarcinoma progression through transcriptional activation of SPHK1. In gastric cancer, elevated HOXC11 expression promotes cell proliferation and migration, accelerates cell‐cycle progression, and is associated with adverse clinical outcomes. Despite these observations, the molecular mechanisms by which HOXC11 governs colorectal cancer growth and metastatic dissemination remain largely unexplored. Defining the role of HOXC11 in CRC progression and its underlying regulatory mechanisms may therefore provide deeper insight into the malignant biology of colorectal cancer and establish a conceptual framework for identifying novel prognostic indicators and actionable therapeutic targets.

In recent years, increasing attention has been paid to the roles of chemokines and their cognate receptors in cancer. Chemokines are a class of cytokines with chemotactic activity, and their cognate receptors are typically expressed by both tumor cells and stromal cells. The binding of chemokines to their receptors contributes to malignant progression by promoting recruitment and activation of inflammatory cells, angiogenesis, and tumor cell proliferation and metastasis [24]. For example, the CXCL12–CXCR4 axis facilitates colorectal cancer metastasis [25]. Binding of the chemokine CCL7 to its receptor CCR1 upregulates SOX18 via activation of the ERK/ELK1 pathway, thereby promoting gastric cancer progression [26]. In hepatocellular carcinoma, HOXB5 activates the CXCL1/CXCR2 axis to drive metastasis and is associated with poor prognosis [27]. CXCL5, a member of the CXC chemokine subfamily, is a ligand for CXCR2; CXCL5–CXCR2 signaling mediates tumor cell migration and invasion as well as neutrophil recruitment [28]. In colorectal cancer, tumor cell‐derived CXCL5 promotes metastasis in a CXCR2‐dependent manner, and CXCL5 overexpression is closely associated with adverse prognosis [29]. Treatment with recombinant CXCL5 enhances CRC cell proliferation and migration/invasion, whereas CXCR2‐neutralizing antibodies abrogate these effects [30]. Collectively, these findings indicate that the CXCL5–CXCR2 axis plays a pivotal role in CRC proliferation and metastasis.

Ca^2^^+^/calmodulin‐dependent protein kinase II (CaMKII) is a major intracellular calcium sensor that converts Ca^2^^+^ signals into downstream protein phosphorylation events, thereby participating in the regulation of cell proliferation, migration, apoptosis, and cell‐cycle progression [31]. CaMKII primarily comprises four distinct isoforms, α, β, γ, and δ. Among them, phosphorylation of CAMK2A at threonine 286 (T286) activates the kinase, enabling it to phosphorylate multiple downstream substrates [31, 32]. In breast cancer, CAMK2A participates in PDSS‐mediated proliferation and metastasis by activating the IL6/JAK/STAT3 signaling pathway [33]. CaMK2A can also promote lung adenocarcinoma progression through activation of EZH2/SOX2 [34]. However, the mechanistic role of CAMK2A in colorectal cancer remains unclear. The NF‐κB family of transcription factors plays a pivotal role in inflammatory responses and innate immunity. Moreover, the NF‐κB pathway is increasingly recognized as a key contributor to tumorigenesis and cancer progression and is aberrantly activated in multiple tumor types [35, 36], prompting us to investigate its role in CRC.

In this study, we validated the biological function of HOXC11 in colorectal cancer proliferation and metastasis and established its clinical relevance in CRC. Mechanistically, we found that HOXC11 activates NF‐κB signaling by transcriptionally upregulating CAMK2A, thereby engaging the CXCL5–CXCR2 axis to promote CRC proliferation and metastasis. Importantly, we identified for the first time a CXCL5–CXCR2–HOXC11 positive feedback loop that plays a critical role in CRC growth and metastatic progression.

## 2 Results

### 2.1 HOXC11 Is Significantly Upregulated and Associated with Poor Prognosis in Human Colorectal Cancer

To identify key HOX family genes that promote colorectal cancer (CRC) progression, we performed bioinformatic analyses of differentially expressed genes (DEGs; FC > 1.5, *p* < 0.05) between CRC tumors and normal intestinal epithelium, as well as between tumors from CRC patients with versus without distant metastasis in TCGA. We intersected these DEGs with HOX family genes (Figure 1A), identifying three upregulated candidates—HOXC11, HOXC12, and HOXC13. Prognostic analysis of TCGA revealed that only high HOXC11 expression in tumors was associated with poor overall survival (OS) (Figure 1B); therefore, we selected HOXC11 as our research subject. Further TCGA analyses showed that elevated HOXC11 expression correlated with unfavorable disease‐specific survival (DSS) and progression‐free interval (PFI) (Figure 1C and Figure S1A). Consistently, analysis of the GSE39582 cohort confirmed that high HOXC11 expression was associated with worse OS in CRC patients (Figure 1D). Next, we measured HOXC11 mRNA expression in 150 paired samples of normal intestinal epithelium, adjacent non‐tumor tissues, and CRC tissues. qRT–PCR showed that HOXC11 was significantly upregulated in CRC tissues (Figure 1E), and HOXC11 mRNA levels were also markedly higher in CRC patients with distant metastasis than in those without metastasis (Figure 1F). Correlation analyses between HOXC11 expression and clinicopathological features (Table S1) revealed that high HOXC11 mRNA expression was positively associated with tumor size (*p* = 0.032), T stage (*p* = 0.008), TNM stage (*p* < 0.001), lymph node metastasis (*p* < 0.001), and distant metastasis (*p* = 0.013), suggesting an important role for HOXC11 in promoting CRC proliferation and metastasis. Consistently, western blotting (*n* = 12) and tissue microarray‐based immunohistochemistry (IHC; *n* = 80) confirmed elevated HOXC11 protein levels in CRC (Figure 1G–I and Figure S1B). Survival analysis of the 80 CRC cases included in the IHC cohort showed that patients with high HOXC11 expression had poorer overall survival (Figure 1J). Analysis of the association between HOXC11 protein levels and the clinicopathological characteristics of patients with CRC revealed that tumor size, tumor stage, lymph node metastasis, and distant metastasis were significantly correlated with HOXC11 expression (Table S7). We also observed that HOXC11 mRNA levels were higher in metastatic CRC tissues than in primary CRC tissues and normal colorectal epithelium (Figure 1K). Collectively, these data indicate that HOXC11 is upregulated in CRC and is associated with adverse clinical outcomes.

**FIGURE 1:**
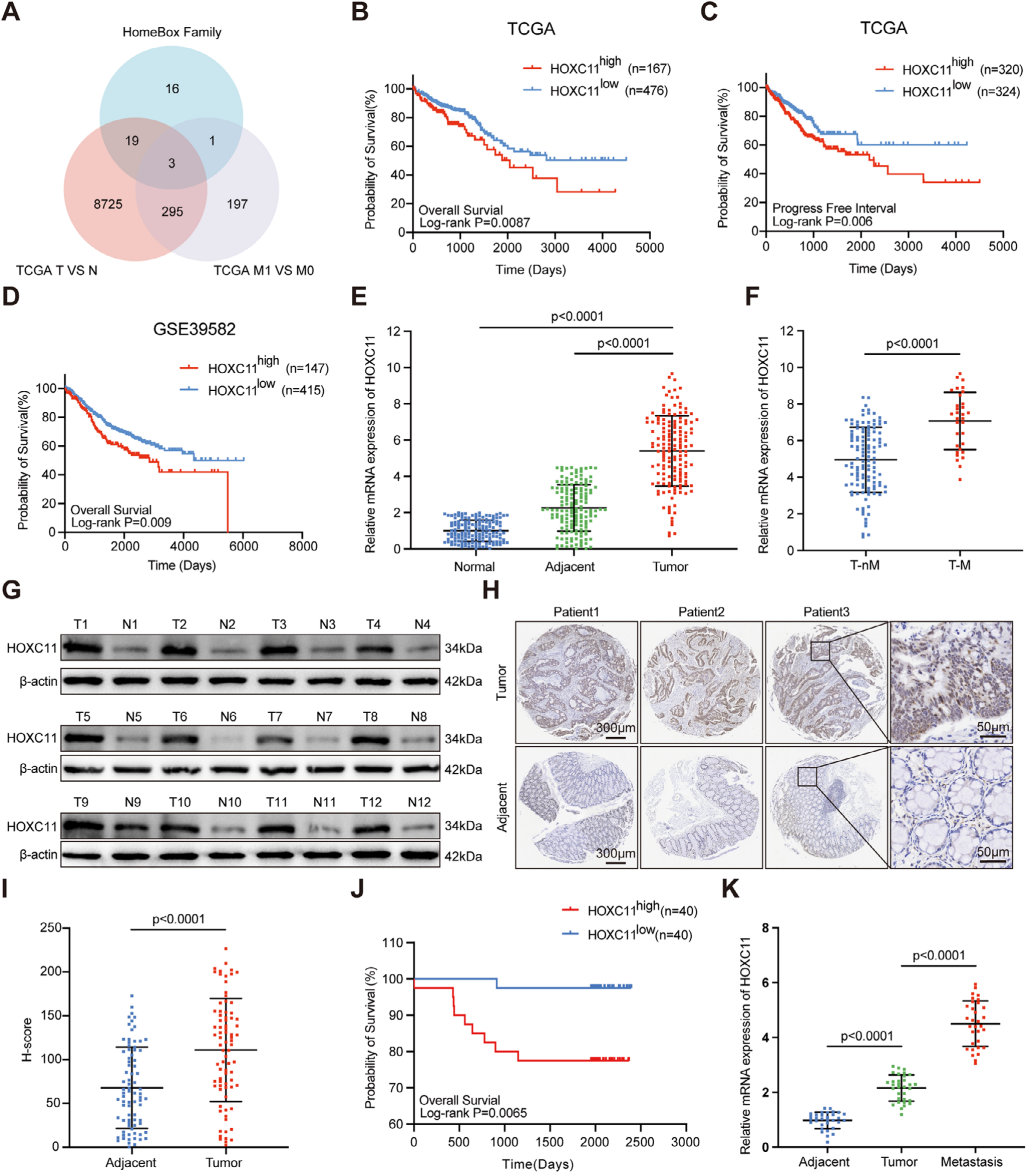
HOXC11 is upregulated in colorectal cancer and is associated with poor prognosis. (A) Venn diagram showing the intersection between HOX family genes and genes upregulated in TCGA CRC tumors versus normal intestinal epithelium, and in tumors from metastatic versus non‐metastatic CRC patients. (B, C) Kaplan–Meier analyses of overall survival (OS) and progression‐free interval (PFI) according to HOXC11 expression in TCGA CRC patients. (D) Kaplan–Meier analysis of OS stratified by HOXC11 expression in the GSE39582 cohort. (E) HOXC11 mRNA levels in 150 paired normal intestinal epithelium, adjacent non‐tumor tissues, and CRC tissues. (F) HOXC11 mRNA levels in tumors from 32 metastatic CRC patients and 118 non‐metastatic CRC patients. (G) Western blot (WB) analysis of HOXC11 protein levels in 12 CRC tissues and matched normal tissues. (H, I) HOXC11 protein expression assessed by immunohistochemistry (IHC) in a tissue microarray (TMA) of CRC patients. (J) Kaplan–Meier analysis comparing OS between HOXC11‐high (n = 40) and HOXC11‐low (*n* = 40) groups. (K) HOXC11 mRNA levels in 32 paired adjacent tissues, primary CRC tissues, and liver metastases. Data are presented as mean ± SD from three independent experiments; *p* < 0.05 indicates statistical significance.

### 2.2 HOXC11 Promotes Proliferation and Metastasis of Colorectal Cancer Cells In Vitro and In Vivo

We quantified HOXC11 mRNA and protein expression in seven CRC cell lines and the normal human intestinal epithelial cell line NCM460 (Figure S1C,F). Based on these profiles, we selected LoVo and DLD‐1 cells with relatively high HOXC11 expression and SW480 and HCT116 cells with relatively low expression for subsequent functional assays. Stable HOXC11 knockdown and overexpression cell lines were generated using lentiviral vectors, and the efficiencies were validated by qRT–PCR and immunoblotting (Figure S1D,E,G). Colony formation assays showed that HOXC11 silencing markedly inhibited proliferation of LoVo and DLD‐1 cells, whereas HOXC11 overexpression enhanced proliferation of SW480 and HCT116 cells (Figure 2A,B and Figure S1H). Transwell and wound‐healing assays consistently indicated that HOXC11 knockdown significantly reduced the migratory and invasive capacities of LoVo and DLD‐1 cells, while HOXC11 overexpression produced the opposite effects (Figure 2C–F and Figure S1I–L). Collectively, these results demonstrate that HOXC11 promotes CRC cell proliferation and metastasis‐related behaviors in vitro.

To investigate the pro‐proliferative and pro‐metastatic roles of HOXC11 in vivo, we established xenograft and liver/lung metastasis models. Subcutaneous injection of LoVo cells stably expressing shControl or shHOXC11‐1, as well as SW480 cells stably expressing oeVector or oeHOXC11, into nude mice was used to generate xenografts. Compared with controls, the HOXC11 knockdown group exhibited significantly reduced tumor volume and tumor weight, indicating that HOXC11 depletion suppresses xenograft growth; conversely, HOXC11 overexpression produced the opposite phenotype. Consistently, immunohistochemistry revealed decreased Ki67 staining in the shHOXC11‐1 group and increased Ki67 staining in the oeHOXC11 group relative to their respective controls (Figure 2G–J). We then constructed liver and lung metastasis models using splenic implantation and tail vein injection, respectively. HOXC11 knockdown markedly diminished fluorescence intensity at metastatic sites and reduced the number of metastatic nodules, whereas the oeHOXC11 group showed significantly higher fluorescence signals and increased metastatic burden compared with controls (Figure 2K–P). These data support a metastasis‐promoting role for HOXC11 in vivo. Collectively, both in vitro and in vivo experiments indicate that HOXC11 facilitates CRC proliferation and metastasis.

**FIGURE 2:**
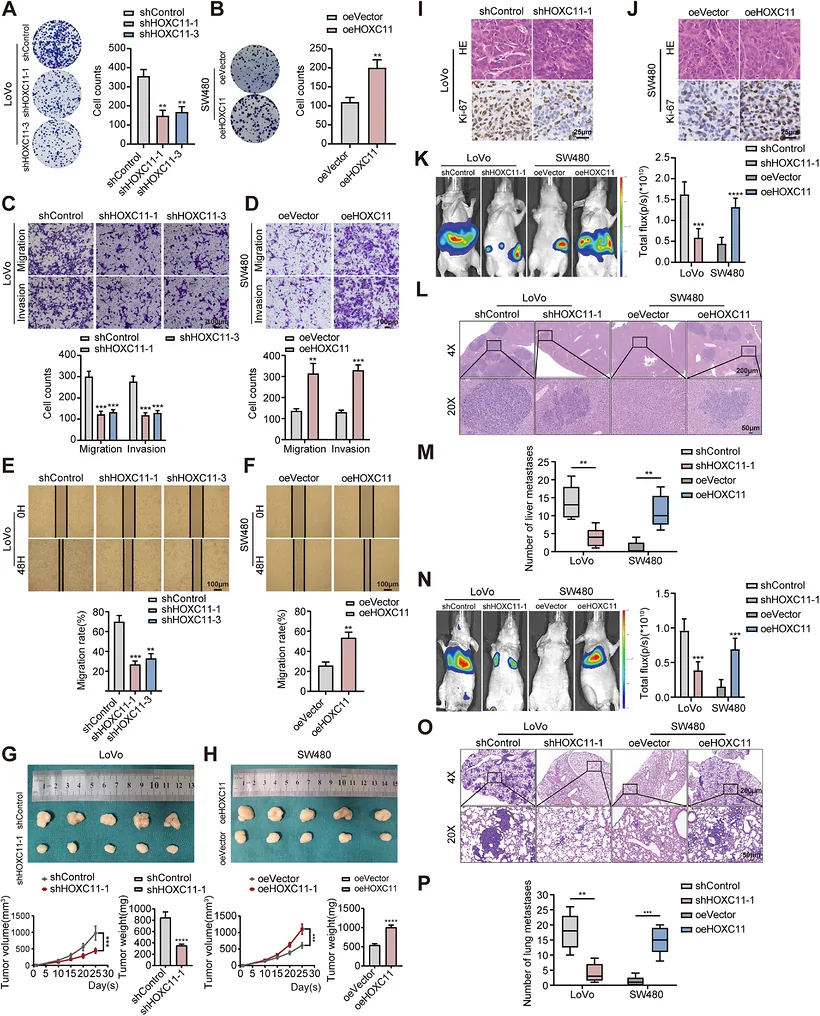
HOXC11 promotes colorectal cancer cell proliferation and metastasis in vitro and in vivo. (A, B) Colony formation assays assessing the effects of HOXC11 on CRC cell proliferative capacity. (C, D) Transwell assays evaluating the impact of HOXC11 on CRC cell migration and invasion. (E, F) Wound‐healing assays assessing HOXC11‐mediated migration and invasion. (G, H) Representative images of nude mouse xenografts, with tumor volume and weight quantified. (I, J) H&E staining and immunohistochemistry (IHC) for Ki67 in xenograft tumors. (K–M) Representative bioluminescence images and quantification of luminescence intensity in the liver metastasis model, together with H&E staining and metastatic nodule counts. (N–P) Representative bioluminescence images and luminescence intensity analysis in the lung metastasis model, together with H&E staining and metastatic nodule quantification. Data are presented as mean ± SD from three independent experiments. **p < 0.01, ***p < 0.001, ****p < 0.0001.

### 2.3 HOXC11 Promotes CRC Proliferation and Metastasis by Upregulating CXCL5 Indirectly

To further elucidate the molecular mechanisms by which HOXC11 promotes CRC proliferation and metastasis, we performed bioinformatic analyses of DEGs between CRC patients with high versus low HOXC11 expression in TCGA. Gene set enrichment analysis (GSEA) indicated that genes enriched in the HOXC11‐high group were associated with “Chemokine receptors bind chemokines” and “Cytokine–cytokine receptor interaction” pathways (Figure 3A,B). We then employed a chemokine/chemokine receptor PCR array to profile chemokine‐related transcripts in SW480 cells stably expressing either oeVector or oeHOXC11. Among the candidates, CXCL5 showed the most prominent upregulation, drawing our attention given its established role in CRC [29, 30]. Immunohistochemistry (IHC) on 80 paired tissue microarrays demonstrated that CXCL5 was highly expressed in CRC tissues (Figure S2A,B), and high CXCL5 expression was associated with poorer overall survival in CRC patients (Figure S2C). Analysis of the association between CXCL5 protein levels and the clinicopathological characteristics of patients with CRC revealed that tumor size, tumor stage, lymph node metastasis, and distant metastasis were significantly correlated with CXCL5 expression (Table S9). Correlation analysis further revealed a positive association between HOXC11 and CXCL5 protein levels (Figure S2D). qRT–PCR and immunoblotting confirmed that HOXC11 knockdown reduced CXCL5 expression in LoVo and DLD‐1 cells, whereas HOXC11 overexpression increased CXCL5 expression in SW480 and HCT116 cells (Figure 3C–E). Furthermore, as CXCL5 functions as a secreted factor, we substantiated these observations through ELISA‐based analysis (Figure S2E,F). To assess the functional contribution of CXCL5 to HOXC11‐driven CRC proliferation and metastasis, we silenced CXCL5 in SW480 and HCT116 cells stably overexpressing HOXC11 and ectopically expressed CXCL5 in LoVo and DLD‐1 cells stably expressing shHOXC11‐1 (Figure 3F). Colony formation assays showed that CXCL5 depletion significantly attenuated the proliferation advantage conferred by HOXC11 overexpression, whereas CXCL5 overexpression rescued the growth inhibition induced by HOXC11 silencing (Figure S2G,H). Transwell and wound‐healing assays similarly demonstrated that CXCL5 knockdown impaired HOXC11‐mediated migration and invasion, while CXCL5 upregulation exerted the opposite effects (Figure S2I–P).

**FIGURE 3:**
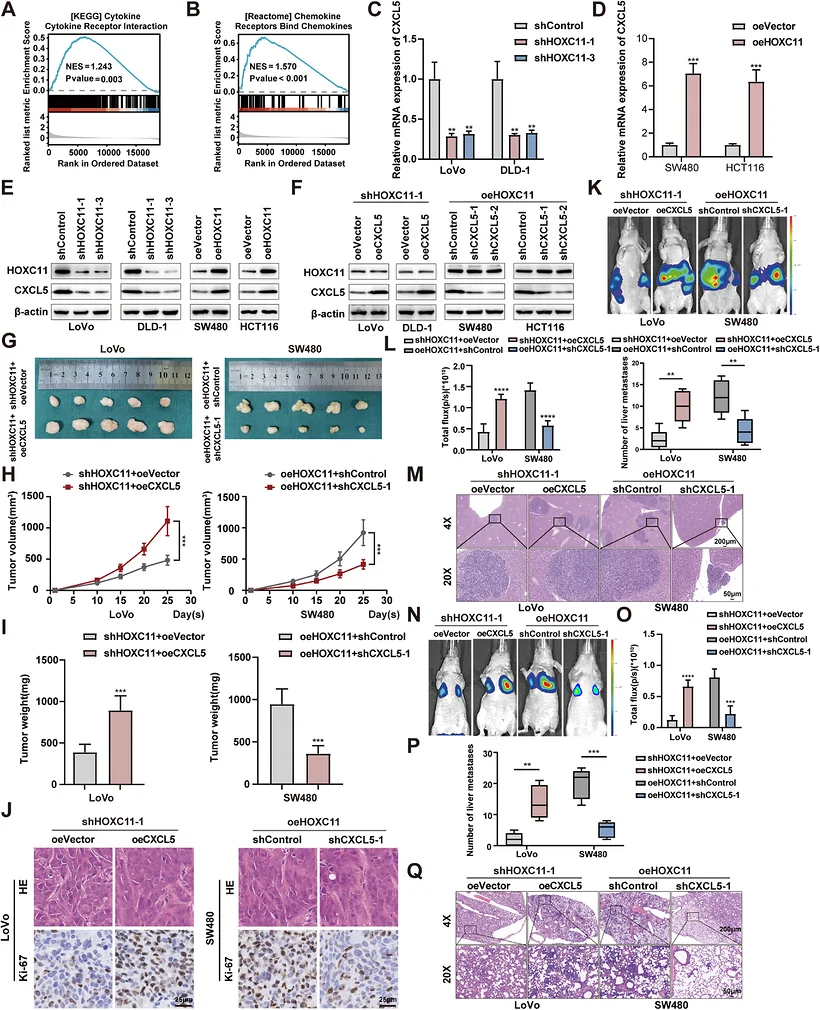
CXCL5 contributes to HOXC11‐mediated CRC proliferation and metastasis in vivo. (A, B) Key signaling pathways identified by KEGG and reactome analyses. (C–E) Changes in CXCL5 mRNA and protein levels following HOXC11 knockdown or overexpression. (F) CXCL5 overexpression or knockdown in the indicated stable cell lines and validation of CXCL5 protein levels. (G–I) Representative images of xenograft tumors derived from stable cell lines with CXCL5 overexpression or silencing, with tumor volume and weight quantified. (J) H&E staining and Ki‐67 IHC staining intensity in xenograft tumors. (K–Q) Liver and lung metastasis models established using stable cell lines with CXCL5 overexpression or knockdown; fluorescence intensity, H&E staining, and metastatic nodule counts are shown. Data are presented as mean ± SD from three independent experiments. **p < 0.01, ***p < 0.001, ****p < 0.0001.

To substantiate the role of CXCL5 in HOXC11‐driven proliferation and metastasis in vivo, we established xenograft and liver/lung metastasis models in nude mice. We found that ectopic CXCL5 expression in LoVo cells stably expressing shHOXC11‐1 rescued the HOXC11 knockdown‐induced reductions in xenograft volume and weight, whereas CXCL5 silencing in SW480 cells stably overexpressing HOXC11 decreased xenograft volume and weight. Consistently, IHC staining showed that Ki67 expression was significantly increased in the CXCL5 overexpression group and markedly decreased in the CXCL5 knockdown group relative to controls (Figure 3G–J). Moreover, in the liver/lung metastasis models, compared with controls, CXCL5 overexpression in LoVo‐shHOXC11‐1 cells increased the number of metastatic nodules and enhanced fluorescence intensity, whereas CXCL5 knockdown in SW480‐oeHOXC11 cells attenuated the HOXC11 overexpression–induced increases in metastatic burden and fluorescence signals (Figure 3K–Q). We next examined whether CXCL5 is a direct transcriptional target of HOXC11. As transcription factors typically regulate gene expression by binding promoter regions, luciferase reporter assays showed that HOXC11 overexpression increased CXCL5 promoter activity (Figure S3A). We identified several putative HOXC11 binding motifs within the CXCL5 promoter using the JASPAR database and generated luciferase reporters harboring mutations in these predicted sites. Notably, mutating the predicted binding sites did not abolish the HOXC11‐induced increase in CXCL5 promoter activity (Figure S3B). These findings suggest that HOXC11 promotes CRC proliferation and metastasis by indirectly upregulating CXCL5 expression.

### 2.4 CAMK2A Is a Direct Downstream Target of HOXC11

To identify direct transcriptional targets of HOXC11, we extracted total RNA from SW480‐oeVector and SW480‐oeHOXC11 cells and performed RNA‐seq. Among the top ten most upregulated genes in the SW480‐oeHOXC11 group (Figure 4A), CAMK2A attracted our attention given its pivotal roles in tumor biology [33, 34]. CAMK2A binds Ca^2^^+^/calmodulin in cells and undergoes autophosphorylation at threonine 286 (T286), enabling phosphorylation of multiple downstream substrates [31, 32]. Immunohistochemistry showed that CAMK2A protein levels were elevated in CRC tissues (Figure S3C,D), and high CAMK2A expression was associated with poorer overall survival in CRC patients (Figure S3E). Analysis of the association between CAMK2A protein levels and the clinicopathological characteristics of patients with CRC revealed that tumor size, tumor stage, lymph node metastasis, and distant metastasis were significantly correlated with CAMK2A expression (Table S8). Moreover, HOXC11 protein abundance positively correlated with CAMK2A protein levels in CRC specimens (Figure S3F). To delineate the contribution of CAMK2A to the malignant behavior of CRC cells, we performed CAMK2A knockdown and overexpression assays (Figure S4A,B), followed by evaluation of cell proliferation, migration and invasion. Depletion of CAMK2A significantly impaired CRC cell proliferation, migration and invasion, whereas ectopic expression of CAMK2A potentiated these malignant properties (Figure S4C–J). Collectively, these results indicate that CAMK2A promotes CRC progression. We subsequently examined whether HOXC11 regulates CAMK2A expression. qRT–PCR and immunoblotting confirmed that HOXC11 knockdown reduced CAMK2A mRNA and protein levels, accompanied by decreased intracellular p‐CAMK2A, whereas HOXC11 overexpression increased CAMK2A mRNA/protein levels and concomitantly elevated p‐CAMK2A (Figure 4B,C). Luciferase reporter assays further showed that HOXC11 upregulation enhanced CAMK2A promoter activity (Figure 4D). Using the JASPAR database, we identified several putative HOXC11‐binding sites within the CAMK2A promoter and generated reporter constructs driven by truncated or mutant CAMK2A promoter fragments. These assays indicated that the −542 to −146 bp region is critical for HOXC11‐mediated activation of the CAMK2A promoter, and mutation of putative binding site 1 within this region attenuated HOXC11‐induced promoter activation (Figure 4E). ChIP assays in CRC cell lines and CRC tissues further demonstrated direct binding of HOXC11 to CAMK2A at the identified motif (Figure 4F,G). Collectively, these results establish CAMK2A as a direct transcriptional target of HOXC11.

**FIGURE 4:**
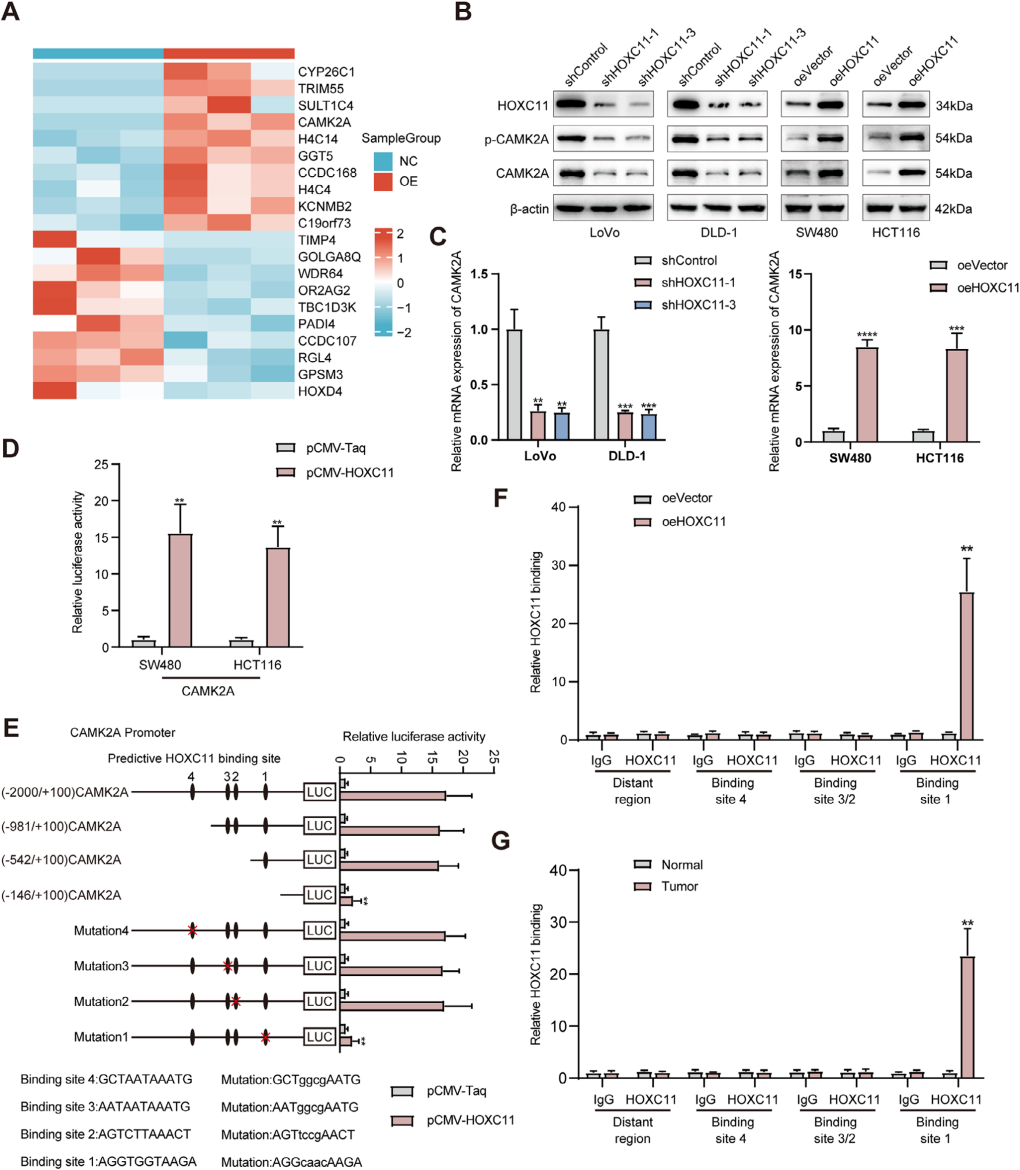
CAMK2A is a direct downstream target of HOXC11. (A) Heatmap showing the 20 most differentially expressed genes between SW480 cells overexpressing HOXC11 and negative control SW480 cells. (B, C) Western blotting (WB) and PCR analyses of the effects of HOXC11 knockdown or overexpression on CAMK2A and p‐CAMK2A expression. (D) SW480 cells were co‐transfected with pCMV‐HOXC11 and a CAMK2A promoter luciferase construct, and promoter activity was assessed using a dual‐luciferase reporter assay. (E) SW480 cells were co‐transfected with pCMV‐HOXC11 and luciferase reporters driven by serially truncated or mutant CAMK2A promoter constructs, and relative luciferase activity was measured. (F, G) ChIP assays showing that HOXC11 directly binds to the CAMK2A promoter in SW480‐oeHOXC11 cells and primary CRC samples. Data are presented as mean ± SD from three independent experiments. **p < 0.01, ***p < 0.001, ****p < 0.0001.

### 2.5 CAMK2A Enhances CXCL5 Expression Through Activation of the NF‐κB Pathway

To delineate the mechanism by which CAMK2A upregulates CXCL5, we silenced CAMK2A in LoVo and DLD‐1 cells and observed a concomitant decrease in CXCL5 protein levels; conversely, CXCL5 expression was increased in SW480 and HCT116 cells overexpressing CAMK2A (Figure 5A,B). Given reports that CAMK2A can activate the NF‐κB pathway [37] and that NF‐κB activation transcriptionally regulates CXCL5 expression [38], we investigated whether CAMK2A induces CXCL5 via NF‐κB signaling. In CAMK2A‐depleted LoVo and DLD‐1 cells, total p65 phosphorylation was reduced, IκBα phosphorylation was decreased while total IκBα protein was increased, and p65 nuclear translocation was inhibited; in contrast, CAMK2A overexpression promoted p65 nuclear accumulation (Figure 5C,D). In CAMK2A‐overexpressing SW480 and HCT116 cells, lentiviral knockdown of p65 or treatment with two NF‐κB inhibitors suppressed the CAMK2A‐driven upregulation of CXCL5 (Figure 5E). Because the IKK complex—primarily composed of IKKα and IKKβ—is critical for IκBα phosphorylation and NF‐κB activation, we next asked whether CAMK2A activates NF‐κB by promoting phosphorylation of IKKα or IKKβ. Notably, IKKβ knockdown in CAMK2A‐overexpressing cells reduced CXCL5 expression, whereas IKKα knockdown had no obvious effect (Figure 5F,G). Consistently, CAMK2A depletion decreased phosphorylated IKKβ levels, while CAMK2A overexpression increased IKKβ phosphorylation (Figure 5H,I). Co‐immunoprecipitation assays further confirmed interactions between endogenous CAMK2A and IKKβ, as well as between exogenously expressed CAMK2A and IKKβ (Figure 5J,K). Together, these findings indicate that CAMK2A activates NF‐κB signaling through IKKβ phosphorylation, thereby promoting CXCL5 expression.

**FIGURE 5:**
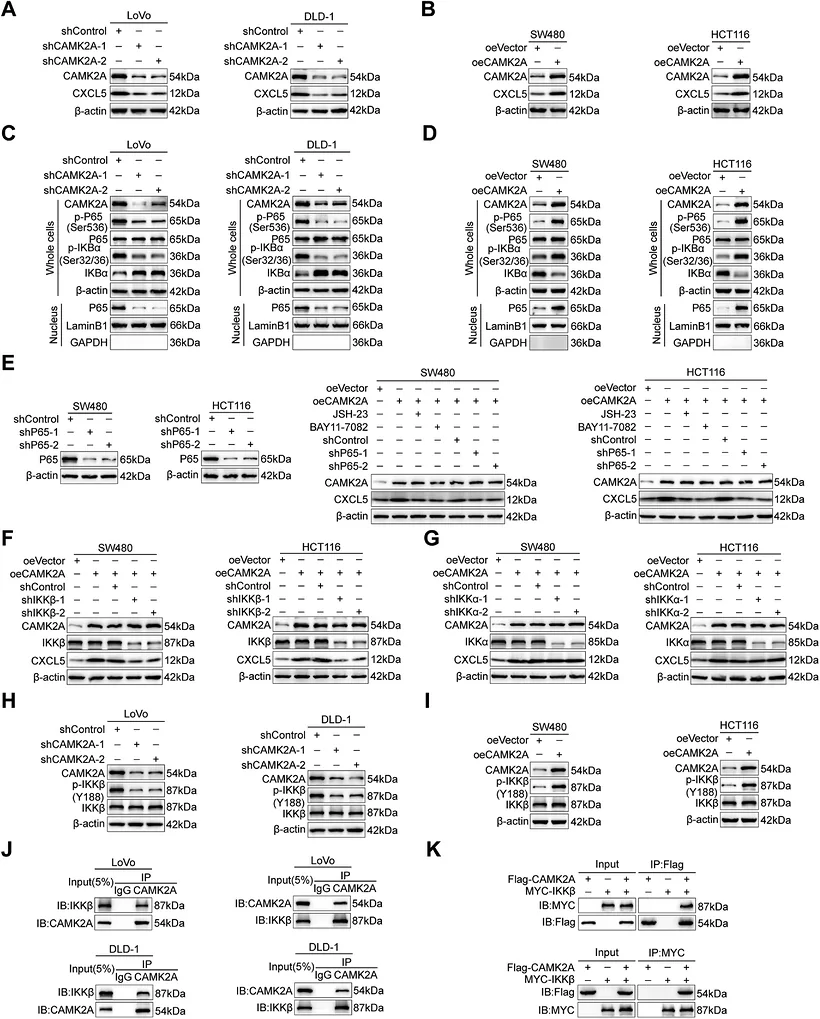
CAMK2A upregulates CXCL5 by activating NF‐κB signaling. (A, B) CRC cells with stable CAMK2A knockdown or overexpression were generated by lentiviral transduction, and CXCL5 protein levels were assessed by western blotting. (C, D) Immunoblot analysis of p‐p65, total p65, p‐IκBα, and total IκBα in lentivirus‐transduced cells, together with assessment of p65 nuclear translocation. (E) Western blotting confirming p65 knockdown by two independent shRNAs in SW480 and HCT116 cells, and analysis of CXCL5 protein levels in CAMK2A‐overexpressing cells following p65 knockdown or treatment with NF‐κB inhibitors. (F, G) CXCL5 protein levels determined by western blotting after knockdown of IKKβ or IKKα in CAMK2A‐overexpressing cells. (H, I) Immunoblot analysis of IKKβ and p‐IKKβ in cells with CAMK2A knockdown or overexpression. (J) Evaluation of the interaction between endogenous CAMK2A and IKKβ. (K) Co‐immunoprecipitation analysis of exogenous CAMK2A and IKKβ in HEK293T cells. All experiments were repeated three times independently. *P* < 0.05 indicates statistical significance.

### 2.6 HOXC11 Knockdown Suppresses CRC Proliferation and Metastasis by Inhibiting CAMK2A‐Mediated Upregulation of CXCL5 Expression

To further investigate whether HOXC11 upregulates CXCL5 via transcriptional activation of CAMK2A, we examined protein levels of CAMK2A, p‐CAMK2A, p‐p65, and CXCL5 in CRC cells with HOXC11 knockdown or overexpression. Immunoblotting showed that HOXC11 silencing decreased CAMK2A, p‐CAMK2A, p‐p65, and CXCL5, whereas HOXC11 overexpression produced the opposite effects (Figure 6A,H and Figures S5A and S6A). To delineate the contribution of CAMK2A to HOXC11‐driven CRC proliferation and metastasis, we ectopically expressed CAMK2A in HOXC11‐depleted LoVo and DLD‐1 cells, which reversed the reductions in p65 phosphorylation and CXCL5 expression caused by HOXC11 knockdown (Figure 6A and Figure S5A). Functional assays in vitro further showed that CAMK2A overexpression rescued the impaired proliferation, migration, and invasion phenotypes resulting from HOXC11 depletion (Figure S5B–F). In xenograft models, CAMK2A overexpression restored tumor volume and weight and markedly increased Ki67 staining (Figure 6B,C). Consistently, in liver and lung metastasis models, HOXC11‐silenced cells overexpressing CAMK2A formed more hepatic and pulmonary metastatic nodules in nude mice and exhibited enhanced fluorescence signals (Figure 6D–G). Conversely, CAMK2A knockdown using two independent shRNAs in SW480‐oeHOXC11 and HCT116‐oeHOXC11 cells suppressed the HOXC11 overexpression‐induced increases in CAMK2A, p‐CAMK2A, p‐p65, and CXCL5 levels (Figure 6H and Figure S6A). Both in vitro and in vivo assays indicated that CAMK2A depletion attenuated the proliferative and metastatic capacities of HOXC11‐overexpressing CRC cells (Figure S6B–F, and Figure 6H–N).

**FIGURE 6:**
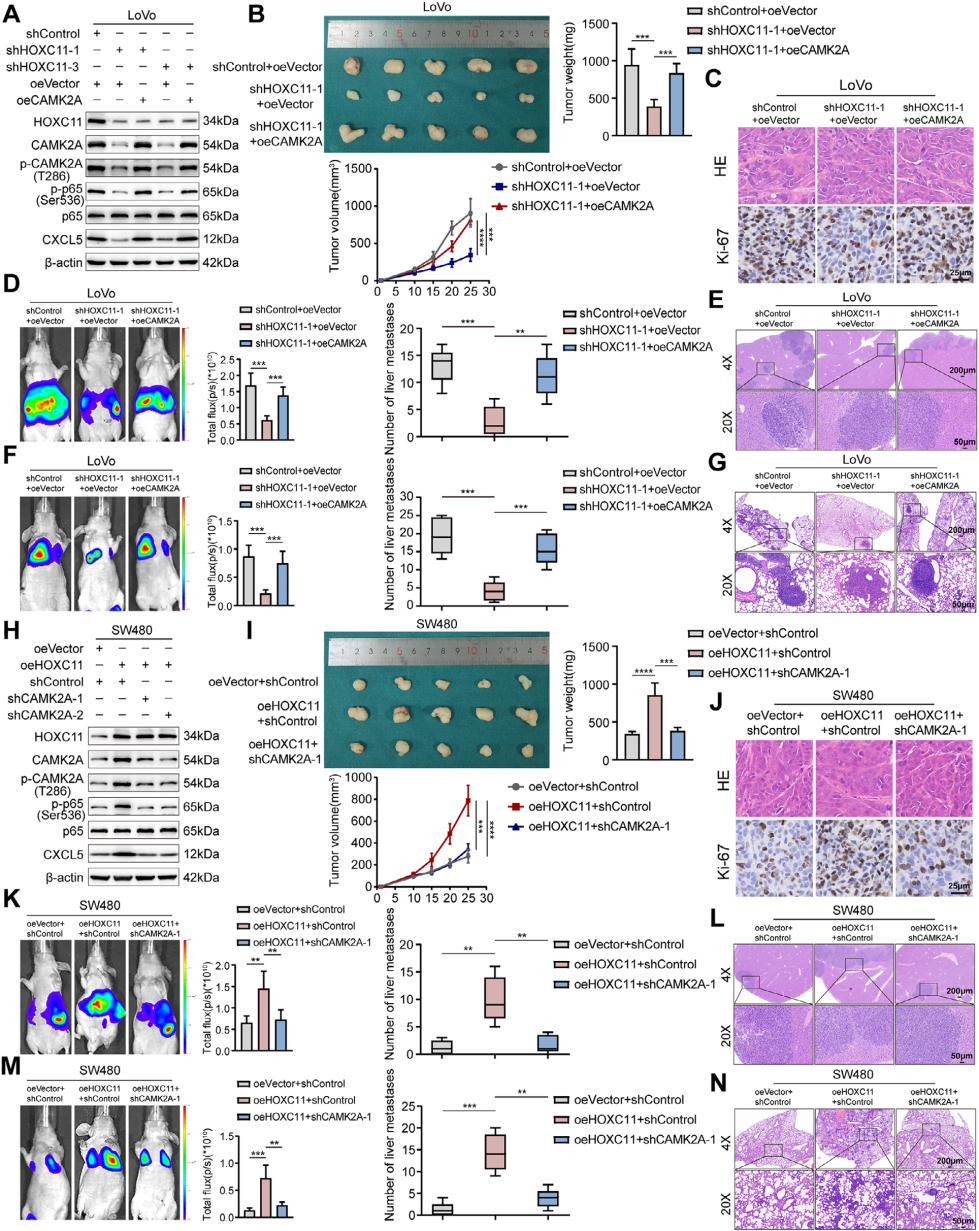
CAMK2A is required for HOXC11‐mediated CXCL5 upregulation. (A) Immunoblot analysis of p‐p65 and CXCL5 levels following CAMK2A overexpression in HOXC11‐silenced LoVo cells. (B) Xenograft assays assessing in vivo proliferative capacity after CAMK2A overexpression in HOXC11‐knockdown LoVo cells. Tumor volume was measured every 5 days, and tumor weight was recorded at endpoint. (C) H&E and immunohistochemistry (IHC) staining of xenograft tumors; IHC was performed to assess Ki67 expression. (D–G) Liver and lung metastasis models evaluating the pro‐metastatic effects of CAMK2A overexpression in HOXC11‐knockdown LoVo cells. (D) Representative in vivo imaging of the splenic implantation liver metastasis model, with quantification of fluorescence intensity and metastatic nodule numbers. (E) H&E staining of mouse livers. (F) Representative in vivo imaging of the tail‐vein injection lung metastasis model, with quantification of fluorescence intensity and metastatic nodule numbers. (G) H&E staining of mouse lungs. (H) HOXC11‐overexpressing SW480 cells were transduced with a negative control or two independent CAMK2A shRNAs, and p‐p65 and CXCL5 levels were assessed by immunoblotting. (I) Representative images of xenografts (*n* = 5 per group) and quantification of tumor volume (measured every 5 d) and tumor weight. (J) H&E and Ki67 IHC staining of xenograft tumors. (K–N) Metastatic potential analysis following CAMK2A knockdown in HOXC11‐overexpressing SW480 cells. (K) Representative in vivo imaging of the splenic implantation liver metastasis model, with quantification of fluorescence intensity and metastatic nodule numbers. (L) H&E staining of mouse livers. (M) Representative in vivo imaging of the tail‐vein injection lung metastasis model, with quantification of fluorescence intensity and metastatic nodule numbers. (N) H&E staining of mouse lungs. Data are presented as mean ± SD from three independent experiments. **p < 0.01, ***p < 0.001, ****p < 0.0001.

### 2.7 The CXCL5–CXCR2 Axis Activates ERK1/2–SP1 Signaling to Induce Upregulation of HOXC11 Expression

Multiple studies have shown that the chemokine CXCL5 promotes tumor proliferation and metastasis [29, 30, 39, 40, 41]. CXCL5 exerts its biological functions by binding to its receptor CXCR2 and activating diverse downstream signaling pathways [42, 43, 44, 45, 46], and the CXCL5–CXCR2 axis can drive transactivation of downstream oncogenes [47, 48]. Importantly, the CXCL5–CXCR2 axis plays a critical role in CRC proliferation and metastasis [29]. Given the pivotal roles of both the CXCL5/CXCR2 axis and HOXC11 in CRC growth and dissemination, together with the aberrantly high expression of HOXC11 in CRC, we sought to determine whether CXCL5–CXCR2 signaling regulates HOXC11 expression in CRC.

We treated SW480 and HCT116 cells, which express relatively low levels of HOXC11, with recombinant CXCL5 for 24 h and found that both HOXC11 mRNA and protein levels were upregulated in a dose‐dependent manner (Figure 7A). Luciferase reporter assays further confirmed that CXCL5 stimulation enhanced the transcriptional activity of the HOXC11 promoter (Figure 7B). We next sought to identify which downstream pathway of the CXCL5–CXCR2 axis is essential for HOXC11 induction. SW480 and HCT116 cells were treated with selective inhibitors targeting candidate downstream pathways, followed by assessment of HOXC11 protein expression. Notably, the ERK pathway inhibitor SCH772984 markedly abrogated CXCL5‐induced HOXC11 upregulation, whereas inhibitors of other pathways showed no appreciable effects (Figure 7C and Figure S7A), indicating that ERK signaling is the critical mediator downstream of CXCL5–CXCR2 for HOXC11 induction.

**FIGURE 7:**
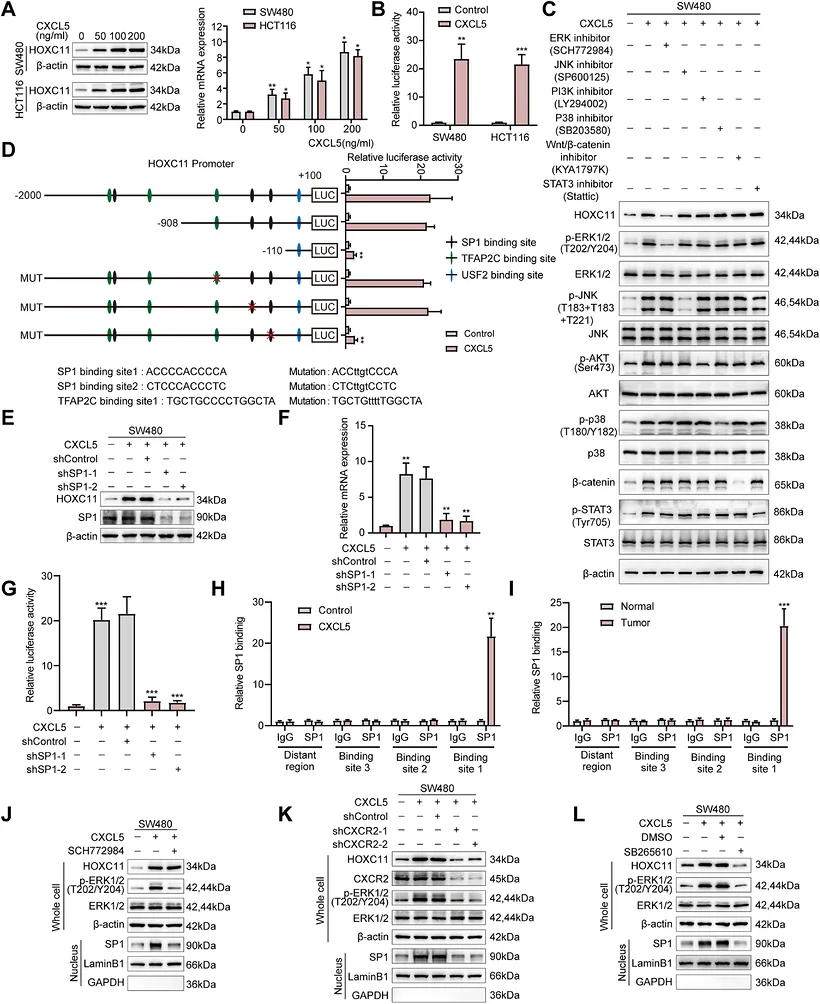
CXCL5 upregulates HOXC11 expression via the CXCR2–ERK1/2–SP1 signaling pathway. (A) SW480 and HCT116 cells were treated with increasing concentrations of CXCL5, and HOXC11 mRNA and protein levels were measured. (B) After treatment with CXCL5 (200 ng mL^−1^) for 24 h, HOXC11 promoter activity was assessed by luciferase reporter assays in SW480 and HCT116 cells. (C) SW480 cells were treated with or without CXCL5 (200 ng mL^−1^) in the presence of pathway‐specific inhibitors for 24 h, followed by western blot analysis of ERK, p‐ERK, JNK, p‐JNK, AKT, p‐AKT, p38, p‐p38, β‐catenin, STAT3, and p‐STAT3. (D) SW480 cells were transfected with luciferase reporters driven by serially truncated or mutant HOXC11 promoter constructs, exposed to CXCL5 (200 ng mL^−1^) for 24 h, and luciferase activity was measured. (E–G) SP1 knockdown attenuated CXCL5‐mediated HOXC11 expression. SW480 cells were transduced with two independent SP1 shRNAs or a negative control and then treated with or without CXCL5 (200 ng mL^−1^) for 24 h; HOXC11 protein (E) and mRNA (F) levels were assessed, and HOXC11 promoter activity was measured by luciferase assays (G). (H, I) ChIP assays showing SP1 binding to the HOXC11 promoter in CXCL5‐treated SW480 cells (H) and primary CRC specimens (I). (J) SW480 cells were cultured with or without an ERK inhibitor and then treated with CXCL5 (200 ng mL^−1^) for 24 h; HOXC11, ERK, p‐ERK, and nuclear SP1 levels were analyzed by western blotting. (K, L) In SW480 cells treated with or without CXCL5 (200 ng mL^−1^) for 24 h, CXCR2 was silenced by shRNA or inhibited with the selective CXCR2 antagonist SB265610, and HOXC11, ERK, p‐ERK, and nuclear SP1 levels were examined. Data are presented as mean ± SD from three independent experiments. *p < 0.05, **p < 0.01, ***p < 0.001.

Next, to identify the transcription factor(s) mediating CXCL5‐dependent transcriptional activation of HOXC11, we analyzed the HOXC11 promoter and performed in silico predictions using transcription factor databases. Intersection of predictions across databases yielded three candidate upstream regulators of HOXC11—SP1, USF2, and TFAP2C (Figure S7B). Predictions of binding sites for these factors within the HOXC11 promoter were made using the JASPAR database. We created a set of luciferase reporter plasmids with truncated or site‐mutated HOXC11 promoter fragments and introduced them into SW480 cells for luciferase testing. These experiments revealed that the −908 to −110 region of the HOXC11 promoter is critical for CXCL5‐dependent promoter activation, and that mutation of the SP1‐binding site within this region markedly reduced CXCL5‐induced HOXC11 promoter activity, whereas mutations in other predicted sites had no obvious effect (Figure 7D). This suggested that SP1 is likely a key trans‐acting factor regulating HOXC11 transcription. Consistently, SP1 knockdown significantly suppressed CXCL5‐induced HOXC11 upregulation and the increase in HOXC11 promoter activity (Figure 7E–G and Figure S7C–E), supporting SP1 as a crucial upstream transcription factor for HOXC11. Subsequent ChIP assays further confirmed the binding of SP1 to the HOXC11 promoter (Figure 7H,I). We added the ERK pathway inhibitor SCH772984 to CXCL5‐treated SW480 and HCT116 cells, which suppressed the CXCL5‐induced increase in nuclear SP1 accumulation and the upregulation of HOXC11 expression (Figure 7J and Figure S7F).

Finally, we examined whether CXCL5 acts in a CXCR2‐dependent manner. CXCL5 stimulation activated the ERK1/2–SP1 axis and increased HOXC11 expression in both SW480 and HCT116 cells; however, lentiviral shRNA‐mediated knockdown of CXCR2 attenuated ERK1/2–SP1 activation, as evidenced by reduced ERK phosphorylation and diminished SP1 nuclear translocation, accompanied by decreased HOXC11 expression (Figure 7K and Figure S7G). Similarly, the CXCR2‐specific inhibitor SB265610 blocked CXCL5‐induced activation of the ERK1/2–SP1–HOXC11 axis (Figure 7L and Figure S7H). Collectively, these results indicate that CXCL5 upregulates HOXC11 expression through activation of the CXCR2/ERK1/2/SP1 signaling axis.

### 2.8 HOXC11 Is Essential for CXCL5‐mediated CRC Proliferation and Metastasis

To further delineate the contribution of the HOXC11–CXCL5 positive feedback circuit, we next examined whether CXCL5 secreted from HOXC11‐overexpressing colorectal cancer cells functionally reinforces malignant traits in CRC cells, and whether HOXC11 is required for CXCL5‐mediated disease progression. Conditioned medium was harvested from HOXC11‐overexpressing CRC cells and subsequently applied to wild‐type CRC cells in co‐culture assays. Exposure to this conditioned medium significantly induced HOXC11 expression in CRC cells (Figure S8A) and concomitantly enhanced their proliferative, migratory, and invasive potential (Figure S8B–F). Importantly, neutralization of CXCL5 largely abrogated conditioned medium‐driven HOXC11 induction and phenotypic malignancy (Figure S8A–F). These results support CXCL5 as a key functional mediator of the pro‐tumorigenic activity present in conditioned medium from HOXC11‐overexpressing CRC cells.

We next investigated whether HOXC11 participates in CXCL5‐mediated CRC proliferation and metastasis. HOXC11‐silenced SW480 and HCT116 cells were treated with CXCL5 (Figure S9A). CXCL5 stimulation markedly enhanced CRC cell proliferation as well as migratory and invasive capacities, whereas HOXC11 knockdown significantly attenuated these CXCL5‐induced phenotypes (Figure S9B–F). In addition, we ectopically expressed CXCL5 in SW480 and HCT116 cells and subsequently transduced these CXCL5‐stable cells with shHOXC11. Immunoblotting confirmed the efficiency of CXCL5 overexpression and HOXC11 knockdown (Figure S10A). CXCL5 overexpression promoted CRC cell proliferation, migration, and invasion, while HOXC11 depletion substantially weakened these enhanced phenotypes (Figure S10B–F). Collectively, these findings indicate that HOXC11 is a critical mediator of CXCL5‐driven CRC proliferation and metastasis.

### 2.9 Combined Treatment with the CAMK2A Inhibitor KN‐93 and the CXCR2 Inhibitor SB265610 Markedly Suppresses HOXC11‐Driven CRC Proliferation and Metastasis

Given that CAMK2A and the CXCL5–CXCR2 axis contribute to CRC progression and play important roles in HOXC11‐mediated promotion of CRC proliferation and metastasis, we investigated whether combined treatment with KN‐93 (a specific CAMK2A inhibitor) [33] and SB265610 (a selective CXCR2 inhibitor) [27] could affect HOXC11‐driven CRC phenotypes. HOXC11‐overexpressing SW480 and HCT116 cells were treated with KN‐93 (10 µmol L^−1^) and SB265610 (100 nmol L^−1^), and changes in relevant proteins were assessed by immunoblotting. Both KN‐93 and SB265610 alone inhibited ERK phosphorylation and SP1 nuclear translocation; notably, their combination produced a more pronounced inhibitory effect than either agent alone (Figure 8A and Figure S11A).

**FIGURE 8:**
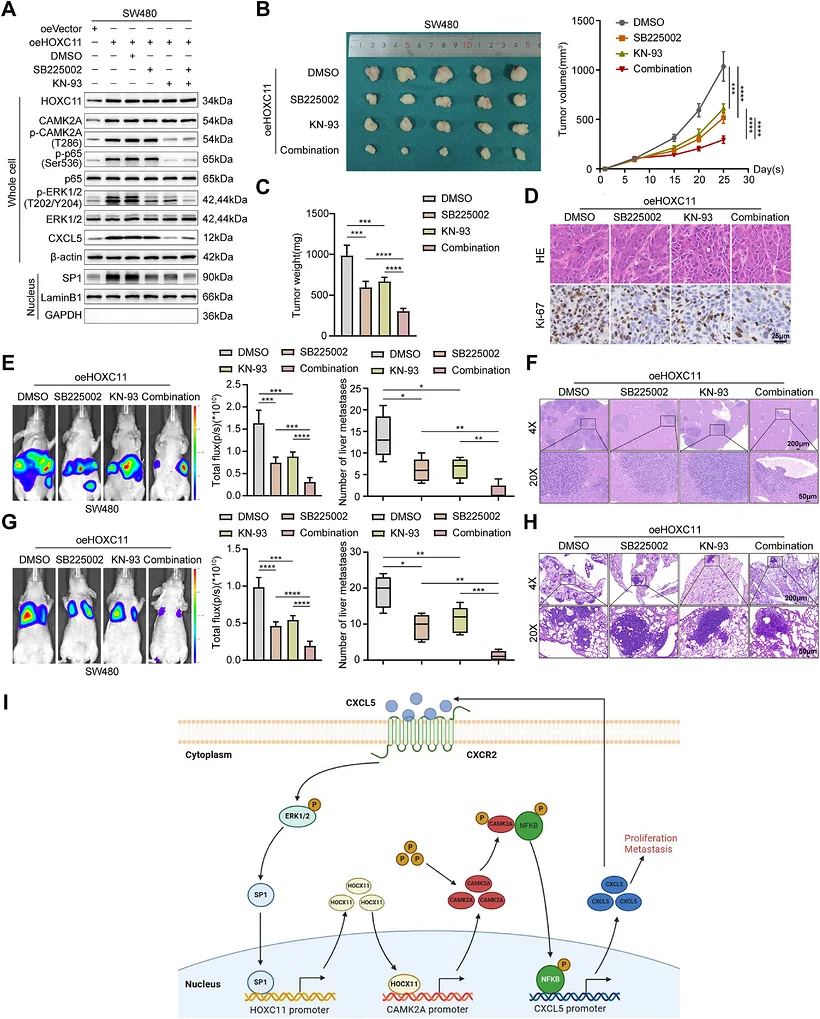
Combined inhibition of CAMK2A (KN‐93) and CXCR2 (SB265610) markedly suppresses HOXC11‐driven CRC proliferation and metastasis in vivo. (A) SW480 cells were treated with KN‐93 (10 µmol L^−1^) and SB265610 (100 nmol L^−1^), alone or in combination, and protein levels of indicated targets were assessed by western blotting. (B–D) In vivo xenograft assays. Compared with the control and single‐inhibitor groups, combined treatment significantly reduced xenograft growth. (B) Representative xenograft images (*n* = 5) and tumor volume measurements (every 5 d). (C) Tumor weight at endpoint. (D) H&E staining and immunohistochemistry (IHC) for Ki67. (E–H) In vivo liver and lung metastasis assays. Compared with the control and single‐inhibitor groups, combined treatment markedly inhibited liver and lung metastasis. (E) Representative in vivo imaging of liver metastases (*n* = 5) with fluorescence intensity quantification and metastatic nodule counts. (F) H&E staining of mouse livers. (G) Representative in vivo imaging of lung metastases (*n* = 5) with fluorescence intensity quantification and metastatic nodule counts. (H) H&E staining of mouse lungs. (I) Schematic model illustrating the pro‐tumorigenic mechanism of HOXC11 in CRC （created using BioRender）.

Next, we evaluated the effects of combined inhibitor treatment on proliferation and metastasis‐related behaviors of SW480‐oeHOXC11 and HCT116‐oeHOXC11 cells. In vitro assays showed that, compared with either agent alone, co‐treatment with KN‐93 and SB265610 significantly suppressed proliferation, migration, and invasion of HOXC11‐overexpressing SW480 and HCT116 cells (Figure S11B–F). Consistently, in nude mouse models, although each inhibitor alone restrained xenograft growth, the combination resulted in markedly smaller tumor volumes and weights, as well as lower Ki67 expression than monotherapy (Figure 8B–D). In liver/lung metastasis models, combined treatment also significantly reduced the number of metastatic lesions and fluorescence intensity, with a greater effect than either single agent (Figure 8E–H). Collectively, these results indicate that dual inhibition with the CAMK2A inhibitor KN‐93 and the CXCR2 inhibitor SB265610 is highly effective in suppressing HOXC11‐driven CRC proliferation and metastasis.

## 3 Discussion

Homeobox (HOX) transcription factors are encoded by a subset of genes within the homeodomain superfamily and play essential roles in multiple aspects of cellular physiology, embryonic development, and tissue homeostasis [4]. HOX proteins are increasingly recognized as important regulators of tumor initiation, progression, and metastasis, and are upregulated in many cancer types. For example, HOXA6 is upregulated in CRC tissues and promotes CRC cell proliferation, migration, and invasion while inhibiting apoptosis [49]. A poor prognosis in colorectal cancer patients is associated with higher expression of HOXA9 protein [50]. In contrast, HOXA5 functions as a tumor suppressor in multiple cancers [17, 18]. In CRC, HOXA5 induces cancer cell differentiation by inhibiting Wnt signaling, thereby impairing the maintenance of tumor stemness [51]. The human HOXB subfamily comprises nine members. Among them, HOXB2 enhances proliferation, migration, and invasion of colon cancer cells by upregulating CCT6A [52], whereas HOXB5 promotes CRC metastasis through transactivation of CXCR4 and ITGB3 [25]. HOXB7 protein levels are significantly associated with advanced Dukes stage, higher T stage, distant metastasis, increased proliferative index, and reduced patient survival [53], suggesting that the HOXB family may play a pivotal role in CRC progression. HOXC10 [54] and HOXD9 [55] have also been shown to be upregulated in CRC tissues and to correlate with disease progression, whereas HOXD8 is thought to suppress CRC progression by acting as an apoptosis inducer [56]. Collectively, the functions of HOX family members in cancer are highly complex, and the mechanisms by which they regulate CRC progression remain incompletely understood and warrant further investigation.

HOXC11 has been reported to play a pivotal oncogenic role in multiple malignancies. For example, in lung adenocarcinoma, HOXC11 promotes disease progression by transcriptionally regulating SPHK1 expression [22]. Several studies have linked HOXC11 to poorer overall survival in colon adenocarcinoma [57], clear cell renal cell carcinoma [58], and gastric adenocarcinoma [23]. In breast cancer, HOXC11 cooperates with steroid receptor coactivator‐1 (SRC‐1) to promote tumor progression by repressing the differentiation marker CD24 and the pro‐apoptotic protein PRKC apoptosis WT1 regulator (PAWR) [59]. Moreover, HOXC11 and SRC‐1 jointly upregulate the calcium‐binding protein S100β, thereby mediating resistance to endocrine therapy [60]. Consistently, increased expression of HOXC11 and SRC‐1, together with their recruitment to the S100β promoter region, has been observed in malignant melanoma [61]. HOXC11 can also activate the androgen receptor via prostaglandin signaling, generating more invasive and endocrine therapy‐resistant breast cancer cells when estrogen signaling is blocked [62]. In our study, integrative analyses of the TCGA cohort and validation in clinical specimens revealed that HOXC11 is upregulated in CRC tissues and may serve as a key regulator of CRC proliferation and metastasis. Survival analyses further indicated that aberrantly high HOXC11 expression is associated with worse prognosis. Both in vitro and in vivo experiments demonstrated that HOXC11 promotes CRC growth and metastatic dissemination. Collectively, HOXC11 is involved in multiple biological processes underlying tumor progression and may represent a promising therapeutic target.

Growing evidence indicates that the emerging roles of C‐X‐C motif chemokine ligand 5 (CXCL5) have been identified across multiple tumor types [63]. In glioblastoma, CXCL5 promotes tumorigenesis and angiogenesis via the JAK–STAT/NF‐κB signaling pathway, and its upregulation is closely associated with adverse clinical outcomes [64]. Collagen‐activated DDR1 enhances CXCL5 expression, leading to a more aggressive phenotype of breast cancer cells. The formation of neutrophil extracellular traps promotes the differentiation and immune infiltration of regulatory T cells in breast cancer [65]. In non–small cell lung cancer, CXCL5 has also been reported to facilitate neutrophil infiltration and suppress the differentiation of anti‐tumor CD8^+ T cells [66]. CAMK2A has likewise been implicated in the regulation of tumor progression in several cancers, including breast and lung cancers [33, 34]. In a glucose‐deprived tumor microenvironment, increased CAMK2A induces SERCA expression by activating NF‐κB signaling, thereby enhancing the resistance of cancer stem cells to metabolic stress [29]. CAMK2A is also involved in Nrf2‐driven esophageal squamous cell carcinoma progression, where Nrf2 promotes radioresistance by targeting CAMK2A to activate autophagy [67]. The role of NF‐κB signaling in cancer has been extensively documented [35, 36], and NF‐κB factors can transcriptionally upregulate multiple chemokines [68]. In the present study, we demonstrate for the first time that CXCL5 participates in HOXC11‐mediated CRC proliferation and metastasis. CXCL5 overexpression reversed the inhibitory effects of HOXC11 downregulation on CRC proliferation and metastasis, whereas CXCL5 knockdown abolished the enhanced proliferative and metastatic capacities conferred by HOXC11 overexpression. Interestingly, HOXC11 did not directly regulate CXCL5; instead, it indirectly promoted CXCL5 upregulation. Using RNA‐seq and experimental validation, we identified CAMK2A as a direct downstream target of HOXC11. HOXC11 transcriptionally upregulates CAMK2A, which promotes IKKβ phosphorylation and activates NF‐κB signaling, ultimately increasing CXCL5 expression. In rescue experiments, CAMK2A silencing suppressed the CXCL5 upregulation induced by HOXC11 overexpression, whereas CAMK2A overexpression restored the reduction of CXCL5 caused by HOXC11 knockdown. In a CRC patient cohort, TMA‐based IHC staining and analyses further confirmed a positive correlation between HOXC11 and CXCL5 protein expression. Collectively, these findings demonstrate that HOXC11 promotes CRC proliferation and metastasis by transactivating CAMK2A to induce CXCL5 upregulation.

However, the mechanism underlying HOXC11 overexpression in CRC remains unclear. It has been reported that chemokine–receptor engagement can activate downstream signaling pathways to upregulate oncogene expression; for example, in gastric cancer, the CCL7–CCR1 axis enhances SOX18 expression by activating ERK/ELK1 signaling [26]. In our study, CXCL5 increased HOXC11 expression by binding its cognate receptor CXCR2 and activating ERK/SP1 signaling, which may explain the aberrant upregulation of HOXC11. HOXC11 overexpression, in turn, promotes CXCL5 secretion, and CXCL5 continuously stimulates CRC cells via autocrine and/or paracrine signaling, thereby establishing a CXCL5/CXCR2/HOXC11 positive feedback loop during CRC progression. This feedback circuit is likely to play an important role in CRC‐associated progression.

Pharmacological modulation of transcription factors involved in pathological processes has long been considered challenging. However, the role of the CXCL5/CXCR2/HOXC11 positive feedback loop in CRC progression cannot be overlooked. The existence of this feedback circuit suggests that blockade of a single molecular component may be insufficient to fully suppress the malignant behavior of CRC cells with high HOXC11 expression, whereas combined targeting of CAMK2A and CXCR2 may more effectively disrupt this pro‐tumorigenic circuit. We focused on the CaMK2A inhibitor KN93 and the CXCR2 inhibitor SB265610, both of which have attracted attention in studies aimed at limiting tumor progression. KN93 exhibits robust anti‐tumor activity in vitro and in vivo [69, 70, 71, 72]. SB265610 is a selective CXCR2 inhibitor that antagonizes CXCR2 through an allosteric mechanism; by binding to the receptor and interfering with G‐protein coupling, SB265610 blocks receptor activation [73], and it has shown anti‐tumor effects in colorectal cancer, prostate cancer, hepatocellular carcinoma, and breast cancer [27, 74, 75, 76]. In this study, we hypothesized that combining KN93 with SB265610 would counteract the HOXC11 overexpression‐driven enhancement of CRC proliferation and metastasis. Our in vitro and in vivo data consistently showed that, compared with control treatment or either agent alone, the combination therapy markedly suppressed HOXC11‐mediated CRC proliferation and metastasis. These findings suggest a potential combination strategy for inhibiting HOXC11‐driven CRC progression.

It should be noted that KN93 and SB265610 currently serve primarily as tools for mechanistic investigation and preclinical validation, and their safety profiles, pharmacokinetic properties, and clinical therapeutic windows require further evaluation. Accordingly, the translational significance of this study lies mainly in proposing an actionable molecular network characterized by “HOXC11 overexpression–CAMK2A/NF‐κB/CXCL5 activation–CXCR2 feedback amplification.” This framework provides a theoretical basis for the subsequent development of CAMK2A/CXCR2‐targeted agents with greater clinical applicability, the identification of patient populations most likely to benefit from combination therapy, and further validation using organoid models, PDX models, and prospective clinical samples.

In summary, we investigated the mechanisms by which the transcription factor HOXC11 regulates CRC proliferation and metastasis. Our data demonstrate that HOXC11 directly upregulates CAMK2A transcriptionally, thereby activating NF‐κB signaling and increasing CXCL5 expression to promote CRC growth and metastatic dissemination. Conversely, CXCL5–CXCR2 signaling induces HOXC11 overexpression via activation of the ERK1/2–SP1 axis, establishing a CXCL5/CXCR2/HOXC11 positive feedback loop that plays an important role in CRC progression.

## 4 Methods and Materials

### 4.1 Tissue Samples and Cell Culture

Written informed consent was obtained from patients and/or their legal guardians for all tissue specimens. Specimens were either rapidly frozen in liquid nitrogen or fixed in formalin right after collection. Procell Life Science & Technology Co., Ltd. (Wuhan, China) supplied the HEK293T cells and the NCM460 normal human colonic epithelial cell line. Human colorectal cancer cell lines such as LoVo, DLD‐1, Caco2, RKO, HT‐29, HCT116, and SW480 were sourced from the Cell Bank of the Chinese Academy of Sciences. Cells were maintained at 37°C in a humidified incubator with 5% carbon dioxide.

### 4.2 Quantitative Real‐Time PCR (qRT‐PCR)

According to the manufacturer's protocol, total RNA was extracted using TRIzol (Invitrogen, USA) and converted into cDNA with HiScript RT Mix (Vazyme, Jiangsu, China). Quantitative real‐time PCR was executed with SYBR Premix Ex Taq (TaKaRa, Dalian, China) following previously described methods. Primer details are provided in Table S2.

### 4.3 RNA Interference and Plasmids

Lentiviruses were produced by Genomeditech (Shanghai, China). Full‐length genes synthesized by Genomeditech were subcloned into lentiviral vectors. All plasmids used in this study were generated by Genomeditech (Shanghai, China). Sequences are provided in Table S3.

### 4.4 In Vitro Cell Assays

Colony formation, Transwell, and scratch wound‐healing tests were performed using methods described in earlier studies [77, 78].

The detailed procedures are described below:

#### 4.4.1 Colony Formation Assay

CRC cells from the treated and control groups were trypsinized, centrifuged, and counted, and then evenly seeded into 6‐well plates (500 cells per well). After a two‐week incubation, colonies were stained with crystal violet, counted, and compared statistically among the groups.

#### 4.4.2 Transwell Migration and Invasion Assays

CRC cells from the treated and control groups were trypsinized, centrifuged, and counted, then uniformly seeded into the upper chambers. After 48 h, cells were stained with crystal violet, and the numbers of migrated and invaded cells were counted.

#### 4.4.3 Wound‐Healing Assay

CRC cells from the treated and control groups were trypsinized, centrifuged, and counted, and 8 × 10^5 cells were evenly seeded per well in 6‐well plates. A scratch of uniform width was generated in the center of each well, and at 0 and 48 h, wound closure was imaged using an inverted microscope and quantified.

### 4.5 Western Blotting

As described before, Western blotting was executed [25]. A complete list of antibodies used in this study is provided in Table S6.

### 4.6 Co‐Immunoprecipitation Assay

Co‐immunoprecipitation (Co‐IP) assays were utilized to analyze the interaction between CAMK2A and IKKβ. Cell lysates were rotated overnight at 4°C with either a specific primary antibody or an IgG control. Immune complexes were incubated for 1 h with Protein A/G magnetic beads (#88802, Thermo Fisher Scientific), washed three times in lysis buffer, eluted in 1× SDS sample buffer, boiled for 10 min, and analyzed by immunoblotting.

### 4.7 Immunohistochemical (IHC) Staining

Immunohistochemistry (IHC) was performed on human colorectal cancer tissue microarrays (TMAs) to determine the expression of HOXC11, CAMK2A, and CXCL5 in CRC. IHC staining was conducted by Servicebio (Wuhan, China). The percentage of positive cells (p_i) was categorized as 0–5%, 6–25%, 26–50%, 51–75%, and 76–100%. The relative expression levels of HOXC11, CAMK2A, and CXCL5 were evaluated using the histochemical score (H‐score), calculated as: H‐score = Σ (p_i × i).

### 4.8 Luciferase Reporter Assay

Dual‐luciferase assays were executed to determine luciferase activity in accordance with the manufacturer's directions (Promega, USA). In culture dishes, transfected cells were lysed with a lysis buffer. The lysates were then transferred to Eppendorf microcentrifuge tubes and centrifuged at maximum speed for one minute. Transfection efficiency was calibrated using Renilla luciferase activity, and the relative luciferase activity was evaluated.

### 4.9 Chromatin Immunoprecipitation (ChIP) Assay

ChIP experiments were conducted following previously reported methods [25]. The detailed procedures are described below:

#### 4.9.1 ChIP in Cell Lines

Around 1 × 10^7 CRC cells were treated with 1% formaldehyde in PBS at 37°C for 10 min, and the process was stopped using 0.125 M glycine. Cells were resuspended in lysis buffer on ice, and chromatin DNA was sonicated to generate fragments of ≈500 bp. The sheared chromatin was then incubated with anti‐HOXC11 or anti‐SP1 antibodies, and using Protein A/G magnetic beads from Thermo Fisher Scientific, USA, antibody‐bound chromatin was isolated. After reverse crosslinking, the DNA was purified with a PCR purification kit provided by Qiagen, USA. Finally, qRT‐PCR was performed to amplify the corresponding binding sites within the promoter regions.

#### 4.9.2 ChIP Assay in Tissue Samples

Surgically resected tissues were washed three times with pre‐chilled PBS (5 min each) and kept on ice in culture medium containing antibiotics and antifungals. Tissues were minced into pieces (<1 mm^3) with sterile blades and digested with DNase I (20 mg mL^−1^) and collagenase (1.5 mg mL^−1^) at 37°C with shaking (250 rpm) for 1 h. The dissociated cells were filtered through a 100‐µm cell strainer, pelleted by centrifugation, and the supernatant was discarded. Red blood cells were lysed for 5 min, and cells were washed with PBS. Subsequent procedures were performed as described for the cell ChIP assay.

Primer details are provided in Tables S4 and S5.

### 4.10 RNA Sequencing (RNA‐seq)

TRIzol was used to extract total RNA from SW480 cells in both the negative control group and the HOXC11 overexpression group, with three biological replicates for each group. RNA quality control, library preparation, and sequencing were performed by Personalbio (Shanghai, China). The gene expression matrix was normalized and log‐transformed using log_2_(FPKM + 1) to reduce differences in sequencing depth and stabilize variance. Significantly differentially expressed genes (DEGs) were defined using uniform thresholds of log_2_FC > 1 and an adjusted *p* value < 0.05.

### 4.11 Animal Models

To establish xenograft and liver/lung metastasis models, six‐week‐old male BALB/c nude mice were utilized. In detail:

#### 4.11.1 Xenograft Tumor Model

A single‐cell suspension of CRC cells at 1 × 10^6 cells/100 µL was prepared from different treatment groups. Each mouse received a 100‐µL subcutaneous injection into the axilla, and measurements of tumor volumes were taken every 5 d. Mice were euthanized 25 days after inoculation, xenograft tumors were excised, and tumor weights were recorded.

To evaluate the effects of CAMK2A and CXCR2 inhibitors on CRC cell proliferation in vivo, mice were randomly assigned to four treatment groups: DMSO, KN93, SB265610, and KN93 + SB265610. Treatment was initiated 10 d after subcutaneous implantation, with daily intraperitoneal administration of KN93 (20 mg kg^−1^) and/or SB265610 (2 mg kg^−1^). Tumor volume and weight were recorded and analyzed.

#### 4.11.2 Liver and Lung Metastasis Models

A model for lung metastasis in vivo was created by injecting luciferase‐labeled cells (1 × 10^6 cells in 100 µL PBS) into the tail vein. To form a liver metastasis model, 1 × 10^6 CRC cells labeled with luciferase were injected into the spleen of anesthetized mice using a 30‐gauge needle. Mice were treated with D‐luciferin (150 mg kg^−1^, i.p.) five weeks later, and metastatic burden in the liver and lungs was quantified using an IVIS100 imaging system. Mice were then euthanized, and the numbers of metastatic nodules in the liver and lungs were counted. To evaluate the effects of CAMK2A and CXCR2 inhibitors on CRC metastasis in vivo, mice were randomly assigned to four treatment groups: DMSO, KN93, SB265610, and KN93 + SB265610. Treatment was initiated 1 week after intrasplenic implantation or tail‐vein injection and administered daily via intraperitoneal injection (KN93, 20 mg kg^−1^; SB265610, 2 mg kg^−1^). Bioluminescence intensity and the number of metastatic nodules were analyzed.

Under the supervision of the Ethics Committee of Nanjing Medical University (IACUC‐2310047), this study was conducted.

### 4.12 Statistics Analysis

GraphPad Prism 9.0 and SPSS 13.0 were used to conduct statistical analyses. Student's t‐test was used to compare differences between two groups, whereas one‐way ANOVA was applied for comparisons among more than two groups. Pearson correlation analysis was used to assess correlations between protein expression levels. Overall survival was analyzed using the Kaplan–Meier method. Associations between gene expression and clinicopathological characteristics were evaluated using the chi‐square test. All experiments were performed at least three times, and data are presented as mean ± standard deviation (SD). P < 0.05 was considered statistically significant.

## Author Contributions

Q.S., Y.S., Y.F., and J.T. were responsible for the overall conception and design of the research. Q.S., H.X., J.Z., and T.W. performed molecular experiments. C.T., C.J., and Z.C. designed and implemented the animal experiments. Q.S. and S.Y. analyzed the experimental results and provided bioinformatics support. Q.S. authored the manuscript. Y.S., Y.F., J.T., and X.W. provided overall supervision of the research, secured funding, and contributed to the interpretation of the results. All authors read and approved the final manuscript.

## Ethics Statement

This study was approved by the Ethics and Research Committee of Nanjing Medical University (2022‐SRFA‐142) and conducted in strict accordance with the Declaration of Helsinki. All participants provided written informed consent prior to the collection of clinical samples. All animal experiments were approved by the Ethics Committee for Animal Experiments of Nanjing Medical University (Ethics number: IACUC‐2305007). All experimental procedures were performed in accordance with the institutional guidelines and regulations for the care and use of laboratory animals.

Clinical trial registration: Not applicable. This study is a basic laboratory research study and does not involve any clinical trial or clinical intervention.

## Conflicts of Interest

The authors declare no conflicts of interest.

## Supporting information




**Supporting File 1**: advs76992‐sup‐0001‐SuppMat.docx.

**Supporting File 2**: advs76992‐sup‐0002‐FigureS1‐S11.zip.

**Supporting File 3**: advs76992‐sup‐0002‐SuppTables.docx.

## Data Availability

The data that support the findings of this study are available from the corresponding author upon reasonable request.
