# Supplementary material for: Transcription Factor HOXC11 Drives Colorectal Cancer Progression and Metastasis via CAMK2A‐Dependent CXCL5 Upregulation
Source: Adv Sci (Weinh). 2026 Aug 19:e76992. Online ahead of print. doi: 10.1002/advs.76992 (PMC13487873; doi:10.1002/advs.76992)
Supplement: Supplementary file 1 [file ADVS-9999-e76992-s001.docx]

**Title:** Transcription factor HOXC11 Drives Colorectal Cancer Progression and Metastasis via CAMK2A-Dependent CXCL5 Upregulation

**Author:** Qingyang Sun, Hengjie Xu, Sheng Yang, Jiahui Zhou, Chuanxin Tian, Hongxu Nie, Chi Jin, Tuo Wang, Zhihao Chen, Junwei Tang, Yifei Feng, Xiaowei Wang, Yueming Sun*

**Supplementary Figures and Legends:**

**Figure S1.** High HOXC11 expression is associated with poor prognosis in CRC patients and promotes CRC cell proliferation and migration/invasion in vitro. **(A)** Kaplan–Meier analysis of disease-specific survival (DSS) according to HOXC11 expression in TCGA CRC patients. **(B)** HOXC11 IHC staining in a tissue microarray (TMA) from 80 CRC patients. **(C, F)** PCR and western blot analyses of HOXC11 mRNA levels in NCM460 cells and the indicated CRC cell lines. **(D–E, G)** PCR and western blot validation of HOXC11 knockdown and overexpression efficiencies. **(H)** Colony formation assays assessing proliferative capacity of DLD-1 and HCT116 cells. **(I–L)** Transwell and wound-healing assays evaluating migration and invasion of DLD-1 and HCT116 cells. Data are presented as mean ± SD from three independent experiments. P < 0.05 indicates statistical significance. *P < 0.05, **P < 0.01, ***P < 0.001, ****P < 0.0001.

**Figure S2.** HOXC11 promotes CRC cell proliferation and migration/invasion by upregulating CXCL5. **(A–C)** CXCL5 protein expression in a CRC tissue microarray (TMA) from 80 patients. **(A)** Representative TMA staining images. **(B)** Statistical analysis of CXCL5 IHC H-scores. **(C)** Kaplan–Meier analysis of overall survival based on CXCL5 expression in the TMA cohort. **(D)** Correlation analysis between HOXC11 and CXCL5 protein expression levels determined by IHC in the TMA. **(E, F)** ELISA of CXCL5 with overexpression or knockdown of HOXC11. **(G, H)** Colony formation assays showing that CXCL5 overexpression enhances proliferation of HOXC11-knockdown LoVo and DLD-1 cells, whereas CXCL5 knockdown suppresses proliferation of SW480-oeHOXC11 and HCT116-oeHOXC11 cells. **(I–L)** Transwell assays showing that CXCL5 overexpression promoted migration and invasion of HOXC11-depleted LoVo and DLD-1 cells, whereas CXCL5 knockdown inhibits migration and invasion of SW480-oeHOXC11 and HCT116-oeHOXC11 cells. **(M–P)** Wound-healing assays showing that CXCL5 overexpression promoted migration/invasion of HOXC11-knockdown LoVo and DLD-1 cells, whereas CXCL5 knockdown reduces migration/invasion of SW480-oeHOXC11 and HCT116-oeHOXC11 cells. Data are presented as mean ± SD from three independent experiments. P < 0.05 indicates statistical significance. *P < 0.05, **P < 0.01, ***P < 0.001, ****P < 0.0001.

**Figure S3.** HOXC11 promotes CXCL5 expression by upregulating CAMK2A. **(A)** SW480 and HCT116 cells were co-transfected with an HOXC11 overexpression construct and a CXCL5 promoter luciferase reporter plasmid, and CXCL5 promoter activity was measured by luciferase assays. **(B)** SW480 cells were co-transfected with pCMV-HOXC11 and luciferase reporters driven by mutant CXCL5 promoter constructs, followed by luciferase activity measurement. **(C–E)** CAMK2A protein expression assessed by IHC in a CRC tissue microarray (TMA) from 80 patients. **(C)** Representative TMA staining images. **(D)** Statistical analysis of CAMK2A IHC H-scores. **(E)** Kaplan–Meier analysis of overall survival stratified by CAMK2A expression in the TMA cohort. **(F)** Correlation analysis between HOXC11 and CAMK2A protein levels determined by IHC in the TMA. Data are presented as mean ± SD from three independent experiments. P < 0.05 indicates statistical significance. **P < 0.01, ***P < 0.001, ****P < 0.0001.

**Figure S4.** High HOXC11 expression is associated with poor prognosis in CRC patients and promotes CRC cell proliferation and migration/invasion in vitro. **(A, B)** Western blot validation of CAMK2A knockdown and overexpression efficiencies. **(C, D)** Colony formation assays assessing proliferative capacity of DLD-1, LoVo, SW480 and HCT116 cells. **(E–J)** Transwell and wound-healing assays evaluating migration and invasion of DLD-1, LoVo, SW480 and HCT116 cells. Data are presented as mean ± SD from three independent experiments. P < 0.05 indicates statistical significance. *P < 0.05, **P < 0.01, ***P < 0.001, ****P < 0.0001.

**Figure S5.** HOXC11 knockdown inhibits CRC cell proliferation, migration and invasion by suppressing CAMK2A-mediated CXCL5 upregulation. **(A)** Immunoblot analysis of p-p65 and CXCL5 levels following CAMK2A overexpression in HOXC11-depleted DLD-1 cells. **(B–D)** Colony formation assays evaluating proliferative capacity of HOXC11-silenced LoVo and DLD-1 cells after CAMK2A overexpression. **(E, F)** Transwell and wound-healing assays assessing migration and invasion of HOXC11-silenced LoVo and DLD-1 cells after CAMK2A overexpression. Data are presented as mean ± SD from three independent experiments. *P < 0.05, **P < 0.01, ***P < 0.001, ****P < 0.0001.

**Figure S6.** HOXC11 overexpression promotes CRC cell proliferation, migration and invasion by enhancing CAMK2A-dependent CXCL5 upregulation. **(A**) HOXC11-overexpressing HCT116 cells were transduced with a negative control or two independent CAMK2A shRNAs, and p-p65 and CXCL5 levels were analyzed by western blotting. **(B–D)** Colony formation assays evaluating proliferation of HOXC11 overexpression SW480 and HCT116 cells after CAMK2A knockdown. **(E, F)** Transwell and wound-healing assays assessing the migratory and invasive capacities of SW480 and HCT116 cells with HOXC11 overexpression after CAMK2A knockdown. Data are presented as mean ± SD from three independent experiments. **P < 0.01, ***P < 0.001.

**Figure S7.** CXCL5 upregulates HOXC11 expression via the CXCR2–ERK1/2–SP1 signaling pathway. **(A)** HCT116 cells were treated with or without CXCL5 (200 ng/mL) in the presence of pathway-specific inhibitors for 24 h, followed by western blot analysis of ERK, p-ERK, JNK, p-JNK, AKT, p-AKT, p38, p-p38, β-catenin, STAT3, and p-STAT3. **(B)** Prediction of transcription factors that directly regulate HOXC11 using AnimalTFDB 3.0, HFtarget, GTRD, and UCSC, and intersection of the predicted results. **(C–E)** SP1 knockdown attenuated CXCL5-mediated HOXC11 expression. HCT116 cells were transduced with two independent SP1 shRNAs or a negative control and treated with or without CXCL5 (200 ng/mL) for 24 h; HOXC11 protein **(C)** and mRNA **(D)** levels were measured, and HOXC11 promoter activity was assessed by luciferase reporter assays **(E)**. **(F)** HCT116 cells were cultured with or without an ERK inhibitor and then stimulated with CXCL5 (200 ng/mL) for 24 h; HOXC11, ERK, p-ERK, and nuclear SP1 levels were analyzed by western blotting. **(G, H)** HCT116 cells treated with or without CXCL5 (200 ng/mL) for 24 h were transduced with CXCR2 shRNA or treated with the selective CXCR2 inhibitor SB265610, and HOXC11, ERK, p-ERK, and nuclear SP1 levels were examined by western blotting. Data are presented as mean ± SD from three independent experiments. **P < 0.01, ***P < 0.001.

**Figure S8.** Conditioned medium from HOXC11-overexpressing CRC cells promotes CRC cell proliferation and metastatic phenotypes. **(A)** Conditioned medium was collected from HOXC11-overexpressing SW480 and HCT116 cells and applied to wild-type SW480 and HCT116 cells for 48h, with or without treatment with a CXCL5-neutralizing antibody (50ng/ml). HOXC11 protein levels were then assessed by western blotting. **(B–D)** Colony formation assays showed that conditioned medium treatment enhanced the proliferative capacity of SW480 and HCT116 cells, whereas CXCL5 neutralization suppressed this effect. **(E–F)** Transwell and wound-healing assays demonstrated that conditioned medium treatment promoted the migratory and invasive capacities of SW480 and HCT116 cells, while CXCL5-neutralizing antibody attenuated the enhanced migration and invasion induced by conditioned medium. Data are presented as the mean ± s.d. from three independent experiments. *P < 0.05, **P < 0.01, ***P < 0.001, ****P < 0.0001.

**Figure S9.** HOXC11 is indispensable for CXCL5-mediated CRC proliferation and metastasis.

**(A**) Stable SW480 and HCT116 cell lines were generated and treated with CXCL5 (200 ng/mL) for 24 h, followed by western blot analysis of HOXC11 protein levels. **(B–D)** Colony formation assays show that CXCL5 treatment promotes proliferation of SW480 and HCT116 cells, whereas HOXC11 deficiency abrogates this effect. **(E, F)** Transwell and wound-healing assays show that CXCL5 enhances migration and invasion of SW480 and HCT116 cells, while HOXC11 deficiency suppresses the CXCL5-induced migratory and invasive phenotypes. Data are presented as mean ± SD from three independent experiments. *P < 0.05, **P < 0.01, ***P < 0.001, ****P < 0.0001.

**Figure S10.** HOXC11 is indispensable for CXCL5-mediated CRC proliferation and metastasis.

**(A)** SW480 and HCT116 cells with CXCL5 overexpression were generated by lentiviral transduction and subsequently subjected to HOXC11 knockdown using shRNA; HOXC11 protein levels were assessed by western blotting. **(B–D)** Colony formation assays show that CXCL5 overexpression promotes proliferation of SW480 and HCT116 cells, whereas HOXC11 deficiency attenuates the proliferative capacity of CXCL5-overexpressing cells. **(E, F)** Transwell and wound-healing assays demonstrate that CXCL5 overexpression enhances migration and invasion of SW480 and HCT116 cells, while HOXC11 loss suppresses the CXCL5-induced migratory and invasive phenotypes. Data are presented as mean ± SD from three independent experiments. *P < 0.05, **P < 0.01, ***P < 0.001, ****P < 0.0001.

**Figure S11.** Combined treatment with the CAMK2A inhibitor KN-93 and the CXCR2 inhibitor SB265610 markedly suppresses HOXC11-driven CRC cell proliferation and metastasis in vitro.

**(A)** HCT116 cells were treated with KN-93 (10 μmol/L) and SB265610 (100 nmol/L), alone or in combination, and the expression of indicated target proteins was assessed by western blotting. **(B)** Compared with the control and single-inhibitor groups, combined treatment significantly reduced cell proliferative capacity. **(C–F)** Compared with the control and single-inhibitor groups, combined treatment significantly decreased migratory and invasive capacities. Data are presented as mean ± SD from three independent experiments. *P < 0.05, **P < 0.01, ***P < 0.001, ****P < 0.0001.
