# Supplementary material for: Transcription Factor HOXC11 Drives Colorectal Cancer Progression and Metastasis via CAMK2A‐Dependent CXCL5 Upregulation
Source: Adv Sci (Weinh). 2026 Aug 19:e76992. Online ahead of print. doi: 10.1002/advs.76992 (PMC13487873; doi:10.1002/advs.76992)
Supplement: Supplementary file 2 [file ADVS-9999-e76992-s002.docx]

**Supplementary Tables**

**Table S1.** Association between HOXC11 expression and clinicopathological characteristics (n = 150).

| **Variable** | **All patients** | **Expression of HOXC11** | | **P value** |
| --- | --- | --- | --- | --- |
|  |  | **High** | **Low** |  |
| All Cases | 150 | 75 | 75 |  |
| Age (years) |  |  |  |  |
| <60 | 41 | 25 | 16 | 0.099 |
| ≥60 | 109 | 50 | 59 |  |
| Gender |  |  |  |  |
| Male | 96 | 44 | 52 | 0.174 |
| Female | 54 | 31 | 23 |  |
| Tumor size (cm) |  |  |  |  |
| <5 | 65 | 26 | 39 | **0.032** |
| ≥5 | 85 | 49 | 36 |  |
| T classification |  |  |  |  |
| T1 + T2 | 60 | 22 | 38 | **0.008** |
| T3 + T4 | 90 | 53 | 37 |  |
| TNM stage (AJCC) |  |  |  |  |
| Stage I+II | 69 | 24 | 45 | **<0.001** |
| Stage III+IV | 81 | 51 | 30 |  |
| Lymph node metastasis |  |  |  |  |
| No | 88 | 31 | 57 | **<0.001** |
| Yes | 62 | 40 | 22 |  |
| Distant metastasis |  |  |  |  |
| No | 118 | 52 | 66 | **0.013** |
| Yes | 32 | 22 | 10 |  |
| CEA (ng/ml) |  |  |  |  |
| <5.00 | 72 | 32 | 40 | 0.191 |
| ≥5.00 | 78 | 43 | 35 |  |

Note: CEA, carcinoembryonic antigen. *P* < 0.05 was considered statistically significant; values in bold indicate *P* < 0.05.

**Table S2.** qPCR primer sequences

| Gene name | Primer type | Primer sequence (5′→3′) |
| --- | --- | --- |
| HOXC11 | Forward Primer | AAAGCCCCAGAGGTTTGTTTTT |
|  | Reverse Primer | AACCTCTGCCCCCAAATAAC |
| CAMK2A | Forward Primer | ACCACTACCTGATCTTCGACC |
|  | Reverse Primer | CCGCCTCACTGTAATACTCCC |
| CXCL5 | Forward Primer | GAGAGCTGCGTTGCGTTTGTTTAC |
|  | Reverse Primer | CCGTTCTTCAGGGAGGCTACCA |
| GAPDH | Forward Primer | GGACCTGACCTGCCGTCTAG |
|  | Reverse Primer | GTAGCCCAGGATGCCCTTGA |

**Table S3.** shRNA sequences

| Name | Sense/antisense sequences | Primer sequence (5′→3′) |
| --- | --- | --- |
| sh-HOXC11 #1 | Sense | CCGGGCGCTGCCCTTATTCGAAATTCTCGAGAATTTCGAATAAGGGCAGCGCTTTTTG |
|  | Antisense | AATTCAAAAAGCGCTGCCCTTATTCGAAATTCTCGAGAATTTCGAATAAGGGCAGCGC |
| sh-HOXC11 #2 | Sense | CCGGCCGAACAATCCTCGAACTAAACTCGAGTTTAGTTCGAGGATTGTTCGGTTTTTG |
|  | Antisense | AATTCAAAAACCGAACAATCCTCGAACTAAACTCGAGTTTAGTTCGAGGATTGTTCGG |
| sh-HOXC11 #3 | Sense | CCGGTCAAGCCTTCGACCGTTTCTTCTCGAGAAGAAACGGTCGAAGGCTTGATTTTTG |
|  | Antisense | AATTCAAAAATCAAGCCTTCGACCGTTTCTTCTCGAGAAGAAACGGTCGAAGGCTTGA |
| sh-CAMK2A #1 | Sense | CCGGGTGCGGAAACAGGAAATTATACTCGAGTATAATTTCCTGTTTCCGCACTTTTTG |
|  | Antisense | AATTCAAAAAGTGCGGAAACAGGAAATTATACTCGAGTATAATTTCCTGTTTCCGCAC |
| sh-CAMK2A #2 | Sense | CCGGGCCAAGGATCTGATCAATAAGCTCGAGCTTATTGATCAGATCCTTGGCTTTTTG |
|  | Antisense | AATTCAAAAAGCCAAGGATCTGATCAATAAGCTCGAGCTTATTGATCAGATCCTTGGC |
| sh-CXCL5 #1 | Sense | CCGGTGAATTGTAGGTGACTATTATCTCGAGATAATAGTCACCTACAATTCATTTTTG |
|  | Antisense | AATTCAAAAATGAATTGTAGGTGACTATTATCTCGAGATAATAGTCACCTACAATTCA |
| sh-CXCL5 #2 | Sense | CCGGCAGACCACGCAAGGAGTTCATCTCGAGATGAACTCCTTGCGTGGTCTGTTTTTG |
|  | Antisense | AATTCAAAAACAGACCACGCAAGGAGTTCATCTCGAGATGAACTCCTTGCGTGGTCTG |
| sh-P65 #1 | Sense | CCGGCGGATTGAGGAGAAACGTAAACTCGAGTTTACGTTTCTCCTCAATCCGTTTTTG |
|  | Antisense | AATTCAAAAACGGATTGAGGAGAAACGTAAACTCGAGTTTACGTTTCTCCTCAATCCG |
| sh-P65 #2 | Sense | CCGGATGGATTCATTACAGCTTAATCTCGAGATTAAGCTGTAATGAATCCATTTTTTG |
|  | Antisense | AATTCAAAAAATGGATTCATTACAGCTTAATCTCGAGATTAAGCTGTAATGAATCCAT |
| sh-IKKα #1 | Sense | CCGGACAGCGTGCCATTGATCTATACTCGAGTATAGATCAATGGCACGCTGTTTTTTG |
|  | Antisense | AATTCAAAAAACAGCGTGCCATTGATCTATACTCGAGTATAGATCAATGGCACGCTGT |
| sh-IKKα #2 | Sense | CCGGGCTGCTCACAAGTTCTATTTCCTCGAGGAAATAGAACTTGTGAGCAGCTTTTTG |
|  | Antisense | AATTCAAAAAGCTGCTCACAAGTTCTATTTCCTCGAGGAAATAGAACTTGTGAGCAGC |
| sh-IKKβ #1 | Sense | CCGGGTATTTCAGACGGCAAGTTAACTCGAGTTAACTTGCCGTCTGAAATACTTTTTG |
|  | Antisense | AATTCAAAAAGTATTTCAGACGGCAAGTTAACTCGAGTTAACTTGCCGTCTGAAATAC |
| sh-IKKβ #2 | Sense | CCGGCAAGGAGAACAGAGGTTAATACTCGAGTATTAACCTCTGTTCTCCTTGTTTTTG |
|  | Antisense | AATTCAAAAACAAGGAGAACAGAGGTTAATACTCGAGTATTAACCTCTGTTCTCCTTG |
| sh-CXCR2 #1 | Sense | CCGGGAAGCGCTACTTGGTCAAATTCTCGAGAATTTGACCAAGTAGCGCTTCTTTTTG |
|  | Antisense | AATTCAAAAAGAAGCGCTACTTGGTCAAATTCTCGAGAATTTGACCAAGTAGCGCTTC |
| sh-CXCR2 #2 | Sense | CCGGGCCACTAAATTGACACTTAAACTCGAGTTTAAGTGTCAATTTAGTGGCTTTTTG |
|  | Antisense | AATTCAAAAAGCCACTAAATTGACACTTAAACTCGAGTTTAAGTGTCAATTTAGTGGC |
| sh-SP1 #1 | Sense | CCGGACCTGGAGTGATGCCTAATATCTCGAGATATTAGGCATCACTCCAGGTTTTTTG |
|  | Antisense | AATTCAAAAAACCTGGAGTGATGCCTAATATCTCGAGATATTAGGCATCACTCCAGGT |
| sh-SP1 #2 | Sense | CCGGAGCCATCATGCCTTGATAAATCTCGAGATTTATCAAGGCATGATGGCTTTTTTG |
|  | Antisense | AATTCAAAAAAGCCATCATGCCTTGATAAATCTCGAGATTTATCAAGGCATGATGGCT |

**Table S4.** HOXC11 binding motifs within the CAMK2A promoter And CHIP Primers

**CAMK2A promoters(-2000~+100)**

>NC_000005.10:c150293130-150290030 Homo sapiens chromosome 5, GRCh38.p14 Primary Assembly

ATGGAAGTAGGGAAGAAAGAGATGACAGGAGCGGGGCTGTGCTGGCCCTGCTCCCAGCAGCCACAGAGGCTGCCCCCGTGGTCCACATCACATACAGGGAGAGAGGTGGCCATCTTCCTGCCTCCATTCCTTCACACCTTCCTCTGAGGGACCTGATGCCAGGGTCTTCCCCATGTGCTCTGCTCACTTCTGAGCATGCTCCATTTCATCAGTGGGAATCGGGAGAGGAAACAGGGGTCAGGCCAGCACCCAGCCACGCAGGACTGTCCCAGTTCAGAACATTAGATTTCCATTAATACAGCCTCATAATCTCACAGCATAGTTGCTTTTTTGTTTTACTGTGAATTTTTAAAACAGCATCATCATTCCTTTGGCACCCGATGAGCTGGAGAGCCTTGTGGTTCCTCTCCTTCTAGCTCTAGTTCTTTGTTCTACAGAAATTGCTGCAAACTAGGCTTAGACAAGGTGATTTCCAAACCATGAGCTAAACTCCAGTGCAGACTAAGGGAACAGTGCCCTGAAGTGCTGGGACTCAAGATGATGGTGTGTGCTTCCGTGATTCAGGGAGTGGTGTGTGAGGGGAGAGGACTTTTGAGTTTCTTGATTATAATTTCCCATTTATTGAGCATTTATAGTGTCAGCCACTGTGCTTGTCTCATTTAATCTACAGAACAGCCTGGAGGTAGGGAATATTATTGACCCAATTATGCAGATGAAGACACAGAGGCACGGAGAAGTTAGGATATTCATCCAAAATCACATAGCCCGTAAGTCGTGGAGTCACCCTAACTACTAAGCAACACTGCCTCTCCTGTCCCTGTGCAATGGCAGGTGGCCTTGGTGGTCACTAAGGGCTCTTCCAGCACTTTCTTGTGTGGAAGCCATCCCCTCCTGGGGGACAGGCTAGAGGGTCTGGGACAGGAGGGTGGCAGTGCAGCCCTCCCCCACCTCCCCACATAGGCCTGTAGCTTGGCCACCGTGTTCTGTGCCCTGCCTGCTGCCCACTGGCTGTGCGGCCCCCAGGCATAGGCCTCTCCACTCTGCCTCTGAATTGCAACGTTGGCGGCAGAAGCGGGAAGCATATTGTGCGCAGAAACAAAACTGAGTGGTTTTCCCTTTTCCCAAAGGTGGTAATGGTGCAATTAGTGGCTAAGCCATTACCCCCCTCCTCCCTTGCTCACTTCCCCTCTTCCCCTCTGCCACCCTCCCTCTCCCACCCTCCCTCTCCTCTCCTGGGAACAGCGGCTGAATGGGTGAGCTCACCCTTTATTCTCCCCCACAGCCCACTCCCAGGAAGAAGGGCAGAATGGGAGGGTTATCCAGATGTCTCCAGGGGGCCCCTGCAAGTAGGGTAGTGAGTCAGGGACTTCATGGGGTTGGAGAATACCAACAAGCAGATAACATGTCCTGCAAGTGAGAGGACTAGCAAGGCAATGTGGAGGTTCAGAGCGGGTGGGCTAGAATCCCTGAGGGTCTGGATGCCTGGATCCTTTTATCCAACCCATGCTCGCTGGGGGCAGGTGCTGGCATGAAGCCTCTGCCCATCAAGCTGCGAAATGGGCCAAGGCACCCTTTTTCCTCAGTGGAGAGGGGAAGGGGTATGCAGGGTCTGAGATGCCAAGCCCAAGCTAGAGTGATGGGGTGGTCACGGGCCAGCTTTAAACCTTCATTTTCCTCCTCTAGAGCCTAGAAAGAGGCCTCCTATTTACATAGTTCCCAAAGGCCTTCACAGCCCTTAGCCCACTCCATCTCCACAGCCTTCCCCTGCAGGGCATCTTCATTCCCCTGTTGTGTAGAAAAGGAGCTGGAGGCTCAGAGAGGGTGAGTGACTTGCCCGTGATCACTCA**GCTAATAAATG(binding site4)**TCCAAAGGCCGGGCATGGTGCCTCACACCTGTAATCCTAGCACTTTGGGAGGCTGAGGCAGGTGGATTGCCTGAGCTCAGGAGTTTGAGACCAGCCTGGGTAACATGGTGAAACCTTGTCTGTACTAAAACACAAAAAATTAGCCAGACGTGGTGGTGTGCGCCTGTAGTCCCAGCTACTTGGGAGGCTGAGGCAGGAGAATCACTTGAACCCAGGAGACGGAGGCTTCAGTGAGCCGAGCTTGCACCACTGTACTCCAGCCTGGGTGACAGAGTAAGACTCTGTCTCCAAAATAAATAAATAAATAAATAAATAATATA**AATAATAAATG(binding site3)**TCCAGGAATCAGAGCTCAAACTCAGATCCTT**AGTCTTAAACT(binding site2)**CCAGTCCCTTTTCTTCCTAACTCCAAGACCTTGGAGTAAGATCTTGTGGCTGTAGGTATGGCTGATGCCCTGAAGAGTTGAAGTTGGCAGGGAAGGTGCCCAGAAAATTTTGGATTGAAGATTTCATGGCAAGTCTCTGGCCAGTGGCCTAGCCCGGGTAAGCCATGCTATGCTCACCTCCCCACAGCCCCCTCTCGCCTTTTTTTTTTTTTTTTTTTTTACCTTGACTGGAAGCACAAGCAGAAACTGGGACATGAGCACCAGGAGACCAGATTTCCATGGTCCCGTTGGGGGCATGGGGTTGGGGAGAGGTTGCAGAGGAGGGCTCTGGAGGGGAGCAACTGTCACAGCTGTGAGAGGTGGGGGTGAGCAGGCAGTCAGGGCTGTTCCCTCCAGAATCCTGGGGTGTCCTCTGCACTTCTGCGCCAAGCTGGAGTGCTAGTGTGATGGACA**AGGTGGTAAGA(binding site1)**GAGCTGAAAGAGCACGAGCATAACAAGAAAGACAGAGGCAGAAGCAAAAAAAAAAAAAAAAAAAAAACAGAGGGCAACAGAGAGACAGTTACAGAGACTACAGTGATCCACAGAGGGAGAGCCATCCCTGTGAATTAGCCATCATTTCCCTGTAAACCTTAGAACCCAGCTGTTGCCAGGGCAACGGGGCAATACCTGTCTCTCTAGAGATGAAGTTGCCAGGGTAACTGCATCCTGTCATTCGTTCCTGGGGACCATCCGGAATGCGGCACCCACTGGCTGTTACCATGGCAACTGCCTTTTTGCCCCACTTAATCCCATCCCGTCTGCTACAAGGGCCCCACAGTTGGAGGTGGGGGAGGTGGGAAGAGAAAAGATCACTTGTGGACAAAGTTTGCTCTATTCC

**Primers used for ChIP in the CAMK2A promoter:**

| **Primer name** | **Primer sequences (5′→3′)** |
| --- | --- |
| distant region sense | TCCTTTGGCACCCGATGA |
| distant region antisense | TTCCCTTAGTCTGCACTGGAGTTT |
| binding site4 sense | GTGAGTGACTTGCCCGTGAT |
| binding site4 antisense | AGGCTGGTCTCAAACTCCTG |
| binding site2/3 sense | GCCTGGGTGACAGAGTAAG |
| binding site2/3 antisense | CATACCTACAGCCACAAGAT |
| binding site1 sense | AGGCAGTCAGGGCTGTTC |
| binding site1 antisense | TTGTTATGCTCGTGCTCTTT |

Note: HOXC11 binding motifs within the CAMK2A promoter. The HOXC11 binding sites are marked in red.

**Table S5.** SP1 binding motifs within the HOXC11 promoter And CHIP Primers

**HOXC11 promoters(-2000~+100)**

>NC_000012.12:53970126-53973226 Homo sapiens chromosome 12, GRCh38.p14 Primary Assembly

TGACTGAGGACACCAAGAGCCAGTGGGGAGGGCCGGATGGCCCTGCTGCAGAGAATTTCAGGTGAAGGCCAGGACTCAAAGCAGATGGAGGGGTCACAGGCACCCACCAGATAAGATACAAATAAGCCACCTGGAGCGGGCTTGACAAGGAAGGGATTGGGGGCCAGGGATGGCTCCTGTACAGGGAGGTATTGATGCTGTGGCCAGAATCCACAGGCTGAGAGGGTGCAGCCTAGGGCAAACATCTCAGCTGAAGCGTCCAGGTAGCCTGGACCCAGGGCTGGGCAGGGAGGCTACTCCAGACCATGGGCATTTTTGCCAGTAAATGTCTAGCTGACCTCAGTGGCCAGGGCCTTCTTTCCTTGACCGTAGCAGCAACTGACAGTTGCTAGAGGTTTTTTGATTTGTTGAAAAACTAAGCCATATCAACACTAGAGAACATTTTAAACTTTATGCCTTACTTTTAAGGGGAGGAATATTACTTTCCAGAAGTCTCTGTTGGGAGACCCCAGCGTTTCCTAGACATCAACTCTTTGACATTCTTTCTTGCATTTCTTTTTTGTTTCAAGGAAATAGGGCCGCCAGCAGCTCCTTGTTGCCGCCTGGCTCACTTTTATGGTGGCCCAGGAATAAACTTTGGTGGTCAAATTCAAGTGCTCAGTCCAGCAGCTGTCTCCTCACAGGATACAGCCCCTACCCCCATCTCAGTCCTGGCTTTGCTGAATCTGCCTTGGGGGATTGGGCCCCACATCACCCTGGAGGGCCAGGAGGGGGAAGGAAGCAGAGCCTTGGGTGGAAGTGGAGAAAGCGGGATCTGAGACTCGAGCCCTCATCCACACCTCTGCAAGGATAGGGGTACCTCTCGCTTCCCTCTTGCTGGTTCCTATCACTGTTTGGGCAGCATTTTGCCTTCAATAAACTCGAGTTTAATGAGGTCAGTGTGAACCCCATTAAAAGCCATAGTCCATAGTTCCCCACCACTATAATTAAAAATGCTGGCCCCATCTCACTCTATAAAAAGGGCTAGAGGAAAAAGGAAACACAGCAGATTTTTCTTTTCATGGCCCCTTACCAACAGAAGTCTCACACTTCAGCAAAGCCCCAATCTAGTTCTTCTTTGCTTCAGTTTAAGATCTGACCAGAACTAAAGGAGTAGCGCCCAGCATTGAGTGCGTAGAGGGGCACCTGAGACCTATCCCTGAGTATTCCAGAACCACCCTGAGAATGGGACCCATGGTGGAGGCAGACTCCACTCAGGTGTCACTTAAATGCTGAGCAAGTTTGAGGAGGCAGGAGCATTGAGGAGGAATGGATTTTAGGGGTTACATATGGAGACAAGCTACAATTTTTTGTGTCCTCCTTATCTGGTTTGGGAGCCGCAGCACCTTATCATGTTTCATCAAGACAAATCCATTCTAATTTCTTTAAGTGGAAACTAATTTTAACTGAGGGTTATGGATGTAAAGAGAGTTCGGGCTATTTTTTGTTTTCCCCTGAGTGTGCATTGGGCTGAGCCCTTGGGGGCTTCGGTTCT**TCCCCTCCTTC(binding site3)**CCAAACAAATTGTGCCCTGGCAGCTCCTCTTTGGATCCAAGGTTGCTTTCAGCTCATGCTGGTTGCTTTTGGCCCCTTGGACCCAGTCCAGAACCTTTCAATAAAGTGATTTTCAGATGCCCACATGTGTCCTGTGGTGCTCAGAGGGATGGAAGGCAAGGGGTTGGGGATGCAGAATGACTGAGCCTACTTCTTGGGCTACAGCTATCCAGGAGGCTGACAGACTTGTAGGAGGGCAGGACAGGGCTGAGAAATAGGAAGGAGGCACTAAGATCTGGTCACAGCCCCAGGGACAGAGGCTGGGGGCTTTGACTAGGGAGTGGCCGCTGGGCCTCCCCTGATGCCACAGCATCCACCGCTCCTTTCCACTGTCTGGTTAGTCTCTTCTGGAGACCCATACCTGGCCCCACCACCCACCTTACAGTCCTCAAAATCATCCAAGGATAAGAACCCCAAACCTAGTCCCCCCCACCATATTCTTCCTGGACCCCTACACTAGGCTTCTTTTCAGCGCACTAGCTGAGGCTGAGCCACTAAGCCAAAGGGCCCTGACATAGAAGATACATGGCTCTCATTCCAATCCTTTTCTTTGGGGTTTGGGATTTGAAAATCCACAGTGGATGTAGCTTTCTTCACATTGATTGACAATAAAGGCAGGCATGAAATATTCACCCAGGGCCACTCTCTCATACTAAATATTTAACACAACATTAGAGAAGGTTTTTCCCAGACTTCGCCAGGCACCGGCTGGGCGGCTGGCGCACTGCTCACCACTCGTTTATGACTAATCTGACTCCCTAGCCAGGGGCAGCACAGCAAGCCAGACAAAGTTGGGACACTGGCTTGCAGCTGGGCTGCCTCACCTTTGGGAAACTTCAGATACTCTTCCGGGAGTCTCTCTAAGGACTCCAGAAAGGTAGTCTTGACACCCAGCTCTGGCCAGGAAAAGAGTGGACTTCTTCAAACTCTTGTGAACAGGCGGTGTCTCTGCGGGTTGGAAGGGTAAGAGTCCCTGAGATTTAAGGGAAAGGATTCACTTCTCTGGGGAAATCAGGCAGTGGGGAAAGAAGTCCTCA**CTCCCACCCTC(binding site2)**CTGGTGCTGCTCACAGAGAGGAGGCAAGACCCCCATACATGAGAGCCCTCCTCCTTCCCCCAATCCTGGGGGAAAAAAGAGCAGCAGCTAGAAAAAAATACAATTACCCCTTCCCAGCCACCACCCTCGCCCATCCCACCGCCC**ACCCCACCCCA(binding site1)**ACCCTCTCTCTCCTCCTTTGCTCGTGCTGTGGTATGGAGAGAAGGGGAGTGACGAGAGAAAAACGAATACAAATCTGCAATTGATTTTCATAATGTTTCTGCGGTGTTTGCAAACCAATCGCCTGAACGTCCCCGATCTTACCTAAGAGAGAACCCCTCCTACGTCTGCGAAGTGCTCCGAACTGATATATGACAATATCTACTTTGGATCACGTGCTCAGAGAGAGAGAGACTAAGACGGATAACGCGTCATCTCGCCTTCCCAAATTTTCCCCCCTCGCTAGACCGGGTCCAAAACCTCCATCCGGAGCCGG

**Primers used for ChIP in the HOXC11 promoter:**

| **Primer name** | **Primer sequences (5′→3′)** |
| --- | --- |
| distant region sense | GGTGGAAGTGGAGAAAGCG |
| distant region antisense | AGGAACCAGCAAGAGGGA |
| binding site3 sense | TTTTAACTGAGGGTTATGGATG |
| binding site3 antisense | TTTGTTTGGGAAGGAGGG |
| binding site2 sense | CTGCGGGTTGGAAGGGTA |
| binding site2 antisense | ACCAGGAGGGTGGGAGTGAG |
| binding site1 sense | AAAAGAGCAGCAGCTAGAAA |
| binding site1 antisense | GCACGAGCAAAGGAGGAG |

**Note:** SP1 binding motifs within the HOXC11 promoter. The SP1 binding sites are marked in red.

**Table S6** Antibodies

| Antibodies | Source |
| --- | --- |
| anti-HOXC11 | Novus |
| anti-CAMK2A | Proteintech |
| anti--p-CAMK2A | Abcam |
| anti-CXCL5 | Abcam |
| anti-P65 | Abcam |
| anti-IKKα | Abcam |
| anti-IKKβ | Abcam |
| anti-IKBα | Proteintech |
| anti- CXCR2 | Proteintech |
| anti-β-actin | Cell signaling technology |
| anti-GAPDH | Proteintech |
| anti- SP1 | Cell signaling technology |
| anti- EKR1/2 | Cell signaling technology |
| anti- p-EKR1/2 | Cell signaling technology |
| anti-JNK | Cell signaling technology |
| anti-p-JNK | Cell signaling technology |
| anti-AKT | Cell signaling technology |
| anti-p-AKT | Cell signaling technology |
| anti-P38 | Cell signaling technology |
| anti-p-P38 | Cell signaling technology |
| anti-β-catenin | Cell signaling technology |
| anti- STAT3 | Cell signaling technology |
| anti- p-STAT3 | Cell signaling technology |
| anti- LaminB1 | Proteintech |
| anti-Flag | Cell signaling technology |
| anti-MYC | Cell signaling technology |

**Table S7.** Association between HOXC11 expression and clinicopathological characteristics (n = 80).

| **Variable** | **All patients** | **Expression of HOXC11** | | **P value** |
| --- | --- | --- | --- | --- |
|  |  | **High** | **Low** |  |
| All Cases | 80 | 40 | 40 |  |
| Age (years) |  |  |  |  |
| <60 | 23 | 11 | 12 | 0.805 |
| ≥60 | 57 | 29 | 28 |  |
| Gender |  |  |  |  |
| Male | 50 | 21 | 29 | 0.065 |
| Female | 30 | 19 | 11 |  |
| Tumor size (cm) |  |  |  |  |
| <5 | 49 | 18 | 31 | **0.003** |
| ≥5 | 31 | 22 | 9 |  |
| T classification |  |  |  |  |
| T1 + T2 | 33 | 12 | 21 | **0.041** |
| T3 + T4 | 47 | 28 | 19 |  |
| TNM stage (AJCC) |  |  |  |  |
| Stage I+II | 43 | 13 | 30 | **<0.001** |
| Stage III+IV | 37 | 27 | 10 |  |
| Lymph node metastasis |  |  |  |  |
| No | 46 | 14 | 32 | **<0.001** |
| Yes | 34 | 26 | 8 |  |
| Distant metastasis |  |  |  |  |
| No | 63 | 26 | 37 | **0.003** |
| Yes | 17 | 14 | 3 |  |
| CEA (ng/ml) |  |  |  |  |
| <5.00 | 49 | 22 | 27 | 0.251 |
| ≥5.00 | 31 | 18 | 13 |  |

Note: CEA, carcinoembryonic antigen. *P* < 0.05 was considered statistically significant; values in bold indicate *P* < 0.05.

**Table S8.** Association between CAMK2A expression and clinicopathological characteristics (n = 80).

| **Variable** | **All patients** | **Expression of HOXC11** | | **P value** |
| --- | --- | --- | --- | --- |
|  |  | **High** | **Low** |  |
| All Cases | 80 | 40 | 40 |  |
| Age (years) |  |  |  |  |
| <60 | 23 | 10 | 13 | 0.459 |
| ≥60 | 57 | 30 | 27 |  |
| Gender |  |  |  |  |
| Male | 50 | 23 | 27 | 0.356 |
| Female | 30 | 17 | 13 |  |
| Tumor size (cm) |  |  |  |  |
| <5 | 49 | 15 | 34 | **<0.001** |
| ≥5 | 31 | 25 | 6 |  |
| T classification |  |  |  |  |
| T1 + T2 | 33 | 10 | 23 | **0.003** |
| T3 + T4 | 47 | 30 | 17 |  |
| TNM stage (AJCC) |  |  |  |  |
| Stage I+II | 43 | 14 | 29 | **<0.001** |
| Stage III+IV | 37 | 26 | 11 |  |
| Lymph node metastasis |  |  |  |  |
| No | 46 | 13 | 33 | **<0.001** |
| Yes | 34 | 27 | 7 |  |
| Distant metastasis |  |  |  |  |
| No | 63 | 27 | 36 | **0.014** |
| Yes | 17 | 13 | 4 |  |
| CEA (ng/ml) |  |  |  |  |
| <5.00 | 49 | 26 | 23 | 0.491 |
| ≥5.00 | 31 | 14 | 17 |  |

Note: CEA, carcinoembryonic antigen. *P* < 0.05 was considered statistically significant; values in bold indicate *P* < 0.05.

**Table S9.** Association between CXCL5 expression and clinicopathological characteristics (n = 80).

| **Variable** | **All patients** | **Expression of HOXC11** | | **P value** |
| --- | --- | --- | --- | --- |
|  |  | **High** | **Low** |  |
| All Cases | 80 | 40 | 40 |  |
| Age (years) |  |  |  |  |
| <60 | 23 | 14 | 9 | 0.217 |
| ≥60 | 57 | 26 | 31 |  |
| Gender |  |  |  |  |
| Male | 50 | 22 | 28 | 0.166 |
| Female | 30 | 18 | 12 |  |
| Tumor size (cm) |  |  |  |  |
| <5 | 49 | 16 | 33 | **<0.001** |
| ≥5 | 31 | 24 | 7 |  |
| T classification |  |  |  |  |
| T1 + T2 | 33 | 11 | 22 | **0.012** |
| T3 + T4 | 47 | 29 | 18 |  |
| TNM stage (AJCC) |  |  |  |  |
| Stage I+II | 43 | 15 | 28 | **0.004** |
| Stage III+IV | 37 | 25 | 12 |  |
| Lymph node metastasis |  |  |  |  |
| No | 46 | 16 | 30 | **0.002** |
| Yes | 34 | 24 | 10 |  |
| Distant metastasis |  |  |  |  |
| No | 63 | 27 | 36 | **0.014** |
| Yes | 17 | 13 | 4 |  |
| CEA (ng/ml) |  |  |  |  |
| <5.00 | 49 | 21 | 28 | 0.108 |
| ≥5.00 | 31 | 19 | 12 |  |

Note: CEA, carcinoembryonic antigen. *P* < 0.05 was considered statistically significant; values in bold indicate *P* < 0.05.
